# Innovative approaches for improving maternal and newborn health - A landscape analysis

**DOI:** 10.1186/s12884-015-0784-9

**Published:** 2015-12-17

**Authors:** Karsten Lunze, Ariel Higgins-Steele, Aline Simen-Kapeu, Linda Vesel, Julia Kim, Kim Dickson

**Affiliations:** Department of Medicine Boston, Boston University, Boston, MA USA; Health Section, UNICEF, 3 United Nations Plaza, New York, NY 10017 USA; Concern Worldwide, 355 Lexington Avenue, New York, NY 10017 USA; GNH Centre Bhutan, Jaffa’s Commercial Building, Room 302, Thimphu, Bhutan

**Keywords:** Innovation, Maternal health, Neonatal health, Continuum of care, LMIC, Review, Implementation

## Abstract

**Background:**

Essential interventions can improve maternal and newborn health (MNH) outcomes in low- and middle-income countries, but their implementation has been challenging. Innovative MNH approaches have the potential to accelerate progress and to lead to better health outcomes for women and newborns, but their added value to health systems remains incompletely understood. This study’s aim was to analyze the landscape of innovative MNH approaches and related published evidence.

**Methods:**

Systematic literature review and descriptive analysis based on the MNH continuum of care framework and the World Health Organization health system building blocks, analyzing the range and nature of currently published MNH approaches that are considered innovative. We used 11 databases (MedLine, Web of Science, CINAHL, Cochrane, Popline, BLDS, ELDIS, 3ie, CAB direct, WHO Global Health Library and WHOLIS) as data source and extracted data according to our study protocol.

**Results:**

Most innovative approaches in MNH are iterations of existing interventions, modified for contexts in which they had not been applied previously. Many aim at the direct organization and delivery of maternal and newborn health services or are primarily health workforce interventions. Innovative approaches also include health technologies, interventions based on community ownership and participation, and novel models of financing and policy making. Rigorous randomized trials to assess innovative MNH approaches are rare; most evaluations are smaller pilot studies. Few studies assessed intervention effects on health outcomes or focused on equity in health care delivery.

**Conclusions:**

Future implementation and evaluation efforts need to assess innovations’ effects on health outcomes and provide evidence on potential for scale-up, considering cost, feasibility, appropriateness, and acceptability. Measuring equity is an important aspect to identify and target population groups at risk of service inequity. Innovative MNH interventions will need innovative implementation, evaluation and scale-up strategies for their sustainable integration into health systems.

**Electronic supplementary material:**

The online version of this article (doi:10.1186/s12884-015-0784-9) contains supplementary material, which is available to authorized users.

## Background

Even with recent global progress towards Millennium Development Goals (MDGs) 4 and 5, an estimated 287,000 women die during pregnancy and childbirth every year [1] and nearly 3 million newborns are estimated to die during their first month of life [2]. Progress in reducing maternal and newborn deaths has been uneven and inadequate. Consequently, disparities persist and reflect barriers and bottlenecks to scaling-up quality health care for women and newborns in low-resource contexts [3, 4]. Proven interventions that reduce maternal and newborn mortality and morbidity are well established [5, 6], yet these essential interventions are not delivered at scale in low- and middle income countries (LMICs) [7]. To increase availability and coverage of these essential interventions, countries must overcome challenges of demand, supply, quality of health care, and the enabling environment [8].

As part of post-2015 planning, efforts at national and global levels are taking stock of achievements and lessons from the MDGs and looking for strategies to further reduce preventable maternal and newborn deaths and disability. In these discussion and analyses, there is frequent reference to innovative approaches as holding potential to improve the quality of services, bring services closer to home, and expand equitable access to essential maternal and newborn health (MNH) [9]. While there is currently no universal definition of innovations in MNH or a systematic description of the landscape of initiatives, tested or currently in implementation, the term ‘innovation’ is frequently used in the domain of MNH to describe new interventions and approaches to service delivery and behaviour change [10].

With an increasing number and variety of innovative approaches for health, the landscape of innovations in the area of MNH has grown more complex [11]. However, the landscape and rationalization of innovative approaches tested in MNH remains poorly understood, as is the evidence of effectiveness associated with these interventions. The objective of this article is therefore to describe the range and nature of approaches considered innovative in MNH care in LMICs, and to analyze the related evidence of their effectiveness or lack herof.

## Methods

We defined a search strategy, informed by a survey of global MNH professionals, to capture the variety of interventions within the continuum of care period from pregnancy to postnatal care. First, we developed a working definition for innovative approaches in MNH. Using an online survey administered to 60 maternal and child health experts globally (including academic, public health, governmental, and private sector stakeholders from implementing and donor organizations), we identified key attributes of innovations in MNH and developed the following working definition to lead our search: “Innovative MNH approaches are novel or newly packaged, potentially scalable interventions, aimed at improving coverage and utilization of quality services across the continuum of maternal and newborn health care to improve MNH outcomes.”

**Fig. 1:**
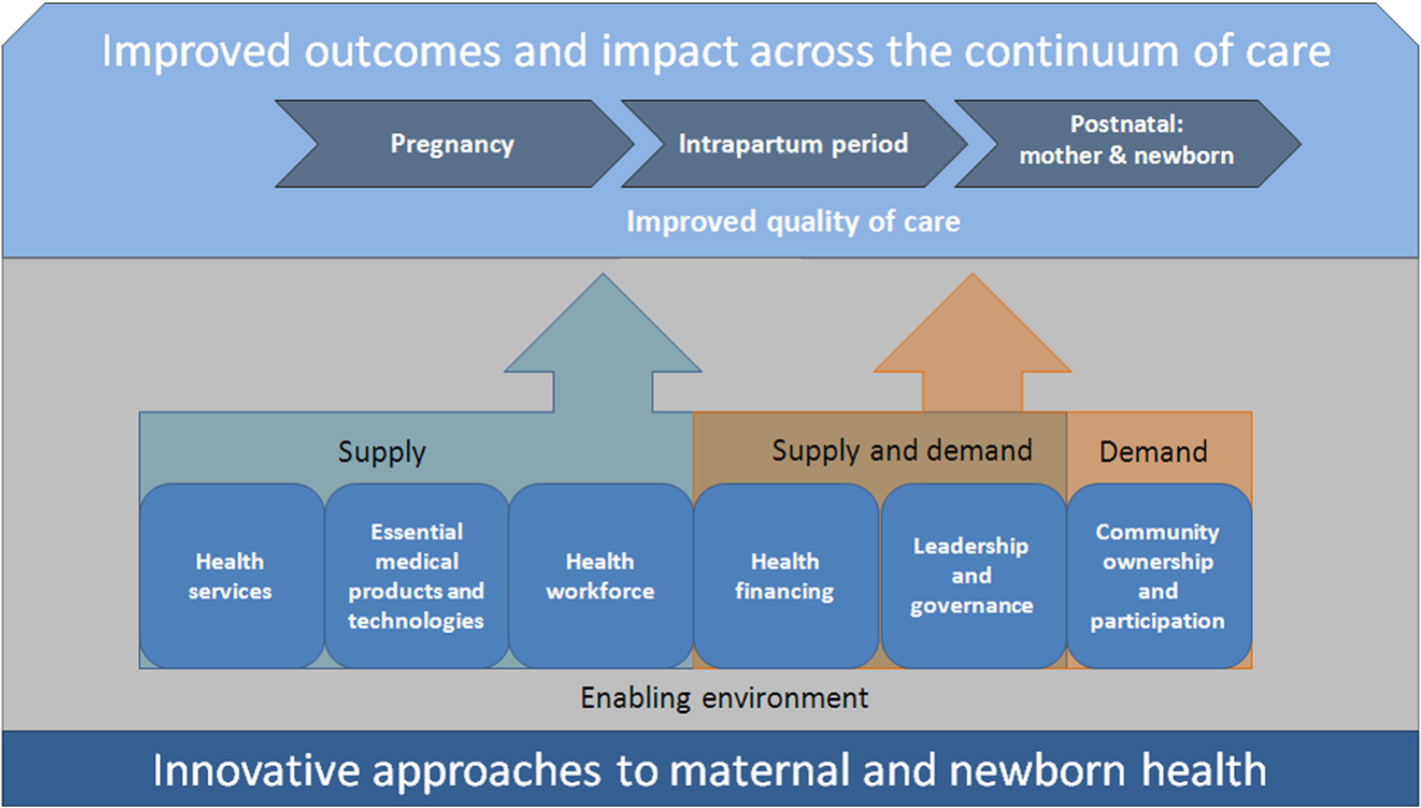
The conceptual framework for describing innovative MNH approaches locates where on the continuum of care interventions target their improvements, and how they aim to achieve these improvements, based on WHO’s health system building blocks and the Tanahashi model of measuring health systems performance

Secondly, we developed a conceptual framework for MNH innovation (Fig. 1) based on the World Health Organization’s (WHO) health system building blocks [12] and the Tanahashi model of measuring health systems performance, [13] centered on the continuum of MNH care [14]. As illustrated in Fig. 1, referring to The Ouagadougou Declaration on Primary Health. Care and Health Systems [15, 16], we modified the WHO building blocks framework to include “Community ownership and participation”. We excluded the building block “health information system” from our analytic framework to somewhat limit the scope of this very broad analysis and to avoid redundancy with recently published reviews and work underway [17–20]. Thirdly, we determined a combination of MNH and innovation terms to search 11 databases (MedLine, Web of Science, CINAHL, Cochrane, Popline, BLDS, ELDIS, 3ie, CAB direct, WHO Global Health Library and WHOLIS). These terms were (for Pubmed): [MeSH] OR (“infant”[All Fields] AND “newborn”[All Fields]) OR “newborn infant”[All Fields] OR “newborn”[All Fields] OR neonat* OR preterm OR premat* OR “mothers”[MeSH Terms] OR “mothers”[All Fields] OR “maternal”[All Fields] OR Matern* OR Mother] AND [“Quality of Care” OR Innovati* OR scale-up OR scaling up OR supply OR demand OR “Program Evaluation”]. We did not specifically conduct a search for gray literature, but included gray literature found in the database search. We searched without language restrictions and included studies in English, French, Spanish and Portuguese.

**Fig. 2:**
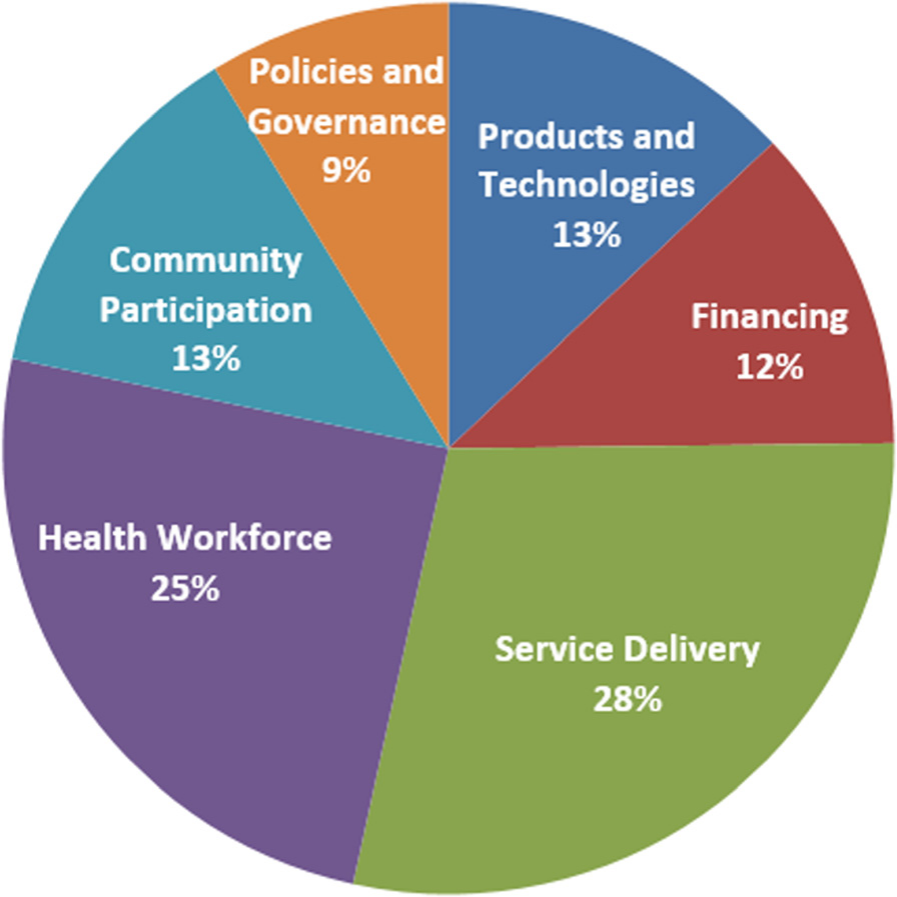
The health system building blocks which innovative MNH approaches aimed to strengthen primarily, *n* = 208

We included studies and gray literature from these databases fitting the following criteria: i) focus on interventions for mothers or newborns (study population) within the continuum of care from pregnancy to the post-natal period (28 days after birth of the neonate), ii) provide a meaningful description of the innovative MNH approach (study interventions) iii) evaluate (see flow chart) or describe novel or newly packaged approaches or ones that were new to a particular target population or context. All peer-reviewed studies were eligible for inclusion, including qualitative studies. To reach to a broad, inclusive overview over the innovation landscape, we included studies regardless of whether they reported outputs, outcomes, or impact data, as long as they provided a description of the intervention. We limited our results to research from LMICs published within the past 10 years. The search was conducted from 15 September to 15 November 2012 (Fig. 2) and followed the PRISMA guidelines [21] where applicable.

Fourthly, we compiled all studies fitting the criteria in a comprehesive inventory (available from authors upon request), which was organized according to our conceptual framework and documented the existing evidence (or lack hereof) on outputs, outcomes, or impact. Finally, two reviewers categorized innovations and graded the evidence of included studies. Study appraisal and grading followed the SIGN Grading System [22] and standards on assessing qualitative research in mixed studies reviews [23–25], as described Additional file 1: Figure S1. The final inclusion and grading of studies was agreed by consensus. Due to the heterogeneity of interventions and study types, we synthesized results descriptively.

## Results

We analyzed 208 innovative approaches reported in 259 studies and reports, including systematic and narrative reviews, randomized controlled trials (RCTs), cluster randomized controlled trials (cRCTs), controlled and uncontrolled pre-post and time series studies, cross sectional studies, and expert perspectives papers (for a complete listing of study results, see Additional file 2: Table S1). Table 1 provides detail on the geographical distribution and types of studies as well as the level of evidence. In order to describe and map innovations into a larger landscape, we categorized findings according to the conceptual framework for MNH innovation we had defined in Fig. 1. We found that innovative MNH approaches relate to all health systems building blocks (Fig. 2, categorized by primary building block), often addressing more than one. Almost all approaches relate to more than one component of the continuum of MNH care – mainly to pregnancy and postnatal care – and address an overlap of demand, supply, or quality. The majority of interventions (72 %) primarily addressed the supply side of health care; only 14 % focused on demand, 10 % on enabling environments (mostly policy initatives), and 4 % on quality of care. Many interventions aimed at serving pregnant women (48 %), often in combination with their newborns (30 %), while others targeted primarily newborns (17 %) or postnatal women and their newborns (5 %).

**Table 1:** Characteristics of innovative approaches to maternal and newborn health care by building block

Health system building block	Geographic region	Setting (urban, rural)	Type of study	Level of evidence^a^
Health service delivery	South Asia (26 %)	*n* = 74	Interrupted time series- 5	SIGN level 1: *n* = 18
	Eastern and Southern Africa (23 % )		Cross-sectional- 4	
	West Africa (14 %)	Rural (34 %)	Pre-post- 7	SIGN level 2: *n* = 1
	East Asia and Pacific (11 %)	Urban (24 %)	Pre-post with control area- 1	
	Latin America and Caribbean (9 %)	Rural and urban (1 %)	Report- 1	SIGN level 3: *n* = 40
	North Africa and Middle East (8 %)	Unspecified (41 %)	Case study- 5	
	Unspecified (9 %)		RCT- 11	SIGN level 4: *n* = 11
			cRCT- 1	
			Qualitative study- 4	B: *n* = 1
			Costing study- 1	
			Literature review- 1	C: *n* = 3
			Mixed methods study- 2	
Medical products and health technologies	South Asia (6 %)	*n* = 35	Pre-post- 4	SIGN level 1: *n* = 6
	Eastern and Southern Africa (11 %)		Narrative review- 9	
	North Africa and Middle East (6 %)	Rural (9 %)	Interrupted time series on	SIGN level 3: *n* = 4
	Unspecified (77 %)	Urban (6 %)	acceptance- 1	
		Unspecified (86 %)	Systematic review- 5	SIGN level 4: *n* = 25
			RCT- 1	
Health workforce	South Asia (31 %),	*n* = 59	Pre-post- 17	SIGN level 1: *n* = 11
	East and Southern Africa (29 %)		Pre-post with control group- 4	
	Latin America and Caribbean (10 %)	Rural (46 %)	Narrative description, feedback- 1	SIGN level 3: *n* = 35
	East Asia and Pacific (7 %)	Urban (24 %)		
	West Africa (7 %)	Unspecified (31 %)	RCT-2	SIGN level 4: *n* = 13
	Central and Eastern Europe (3 %)		cRCT- 1	
	Unspecified (14 %)		Systematic review- 6	
			Case study- 1	
			Cross-sectional- 6	
			Cross-sectional survey on satisfaction- 1	
			Cross-sectional survey with control group- 1	
			Costing study- 1	
			Narrative review- 13	
			Report- 2	
			Interrupted time series- 1	
			Study protocol- 1	
Health financing	South Asia (41 %)	*n* = 32	Case study- 2	SIGN level 1: *n* = 7
	West and Central Africa (28 %)		Interrupted time series and	
	East and Southern Africa (19 %)	Rural (25 %)	qualitative- 1	SIGN level 2: *n* = 1
	East Asia and Pacific (13 %)	Urban (6 %)	Protocol- 3	
		Rural and urban (59 %)	Cross sectional- 3	SIGN level 3: *n* = 17
		Unspecified (9 %)	Cross sectional and qualitative- 1	
			RCT- 1	SIGN level 4: *n* = 4
			cRCT- 1	
			Pre-post with control- 2	A: *n* = 1
			Pre-post- 1	
			Qualitative- 3	B: *n* = 1
			Non-random controlled trial- 2	
			Non-random controlled quasi experimental trial- 1	C: *n* = 1
			Interrupted time series- 7	
			Interrupted time series with controls; and qualitative- 1	
			Systematic review- 1	
			Narrative review- 2	
Community ownership and participation	South Asia (66 %)	*n* = 35	cRCT- 8	SIGN level 1: *n* = 9
	Eastern and Southern Africa (14 %)		Narrative review- 6	
	East Asia and Pacific (11 %)	Rural (86 %)	Qualitative study- 4	SIGN level 3: *n* = 13
	Latin America and the Caribbean (3 %)	Urban (11 %)	Systematic literature review- 1	
	West and Central Africa (3 %),	Unspecified (3 %)	Pre-post with control- 2	SIGN level 4: *n* = 9
	Unspecified (3 %)		Pre-post- 6	
			Commentary- 1	B: *n* = 1
			Cross sectional survey and qualitative- 1	C: *n* = 3
			Study protocol- 2	
			Cross sectional study- 2	
Leadership and governance	South Asia (38 %)	*n* = 24	Pre-post- 1	SIGN level 3: *n* = 5
	East Asia and Pacific (17 %)		Pre-post with comparison areas- 1	
	Eastern and Southern Africa (13 %)	Rural (33 %),		SIGN level 4: *n* = 17
	Latin America and the Caribbean (13 %)	Urban (4 %)	Narrative review- 3	
	North Africa and Middle East (8 %)	Unspecified (63 %)	Policy analysis- 7	B: *n* = 2
	West and Central Africa (8 %),		Case study- 10	
	Unspecified (4 %)		Report- 1	
			Qualitative study- 1	

^a^See Additional file 1: Figure S1

Most studies on innovative approaches included in this review occurred in Africa (34 %), South Asia (32 %) or East Asia (9 %), and only a few were from Latin America (7 %) or Central/Eastern Europe (1 %) (17 % did not specify a country or region). Among the studies that specified the setting in which they were carried out, 35 % were conducted in rural settings, 15 % in urban environments and 13 % in both. The vast majority of published studies were observational studies or expert opinion papers (75 %).

The following sections describe the landscape of MNH innovations by primary health system building block, highlighting key approaches and their existing evidence as substantiated by this review. Table 2 summarizes these results.

**Table 2:** Summary of innovative approaches to maternal and newborn health care by building block

Health system building block	Innovative Approaches/Strategies
Health service delivery	Quality improvement
	• Management and leadership skills development activities • Safe childbirth checklist, a standardized protocol for MNH care • Implementation of redesigned care model/protocol based on selected evidence-based recommendations and women’s views • Collaborative quality improvement of a network of sites working together • Comprehensive intervention packages based on quality improvement approaches (including certifications, delivery of services, incentives, promotion, etc.) • UNICEF Safe motherhood programme • Special care newborn units to provide high quality care • Infection control programme to reduce nosocomial infections • Package of MNH interventions at institutional level • Mental health care for pregnant women using existing primary care resources • Provision of equipment and training to facilities • Community education on maternal health • Application of quality of care model from family planning to EmOC
	Skin-to-skin care / kangaroo mother care
	• Community-based kangaroo mother care • Kangaroo mother care implementation tool to monitor progress • Implementation of kangaroo mother care in government hospitals • Use of facilitation to implement kangaroo mother care in hospitals
	MNH nutrition
	• New micronutrient supplementation programs (e.g. zinc, iron, calcium) • Positive deviance approach to improve antenatal nutrition
	Breastfeeding
	• Innovative promotion strategies (e.g. postnatal visits, counselling by community volunteers, mass media) and delivery systems (e.g. baby-friendly hospitals, peer facilitators) including mainstreaming breastfeeding into the scale-up of MNH
	Prenatal care
	• Maternity waiting homes, some combined with MCH services and income generation activities • Yoga for high risk pregnancies • Education for first time childbearing women • Group prenatal care
Medical products and health technologies	Maternal
	• Non-pneumatic anti-shock garment to stabilize and resuscitate hypovolemic shock • Automated blood pressure devices for low resource settings • Single use obstetric emergency kits • Misoprostol for community-based use, storage and application system for oxytocin delivery and balloon condom catheter to treat intractable uterine bleeding • Foilized polyethylene pouch to store neviparine • Low-cost, low-tech devices: portable OB ultrasound and Doppler, simplified partograph, vacuum delivery/EmOC devices, birth simulator, cell-phone-based malaria diagnostics, hemoglobinmeter, EmOC transporter (eRanger) • Clean delivery kits
	Neonatal
	• Low-cost devices: ventilator support, temperature measurement, pulse oximeter and phototherapy • Devices to prevent PMTCT (e.g. breastfeeding shields) • Application of chlorhexidine for umbilical cord care • Topical application of emollients to reduce nosocomial infections and mortality • Thermoprotection mechanisms: cot-nursing using heated water-filled mattress, infant warmers, wraps and foils
Health workforce	Training
	• E-learning via internet and phone text messages • Training of community health worker cadres in tasks previously not assigned: antenatal care, safe delivery, neonatal resuscitation, essential newborn care and PMTCT care, IMNCI • Low-technology obstetric and neonatal resuscitation simulation training (e.g. Helping Babies Breathe Programme) • Training programs/courses for trainers and providers in antenatal care, EmOC, essential newborn care and neonatal resuscitation: Making Pregnancy Safer, Promoting Effective Perinatal Care, WHO Essential Newborn Care, acute care of at-risk newborns, Perinatal Continuing Education Programme, Essential Surgical Skills Emergency MCH Programme • Partnering international professional organizations for training of providers • Training TBAs in antenatal care, safe delivery, neonatal resuscitation and essential newborn care, use of delivery mat and misoprostol • Training of nurses: quality improvement tools, oxytocin use
	Task-shifting to non-physicians
	• Non-physician clinicans to provide EmOC • Anaesthesia services provided by mid-level cadres • NICU newborn aides to help staffing problems • Pictorial job aids used by providers
Health financing	Enhancing demand for MNH services
	• Conditional cash transfers • Cash incentives for skilled delivery at facility • Vouchers for maternal health services and related costs (e.g. transport costs and cash payment for delivery at facility) • Community-based health or obstetric insurance • Abolition or reduction of user fees
	Incentives for health workers to increase supply and quality of services
	• Performance-based payment • Free reimbursement for training and costs
Community ownership and participation	Women’s groups and community-based intervention packages
	• Women’s groups convened by female facilitators to identify problems and formulate solutions • Female community health worker outreach • Community/home-based intervention packages including pregnancy, delivery and ENC components
	Linkage between community and facility
	• Integration of newborn care into existing community-based package and national health system • Creating a network of providers/CHWs
	Community mobilisation
	• Community-based quality improvement process involving learning and problem-solving cycle • Home-based care and linkages to facility based services including distribution and use of misoprostol, recognition of danger signs, improvements in transport • Community participatory learning activities • Positive deviance behavior change activities
Leadership and governance	Partnerships
	• Public-private partnerships, international/regional partnerships and inter-agency task teams to create capacity for MNH care
	National MNH policies
	• Health system reforms • Use of research, data and policies to develop community-based newborn care package/national newborn strategy and influence high-profile champions to act • Integration of skilled birth attendance into national plan/policy • Increase in political commitment • Rights-based programming and micro-planning strategy to increase access, coverage and quality of MNH care • Use of situation analysis to develop newborn action plan

### MNH service delivery

The majority of currently published literature reflects that innovative approaches in MNH care aim at improving health service delivery along the continuum of care and ultimately MNH outcomes. Service delivery innovations often combine their approaches with elements from other building blocks, e.g. with innovative financing models, training of providers, and new technologies. Studies evaluated both facility-based and community- or family-based innovative approaches in implementation, organization or quality of MNH care.

We included quality improvement projects where they were described as innovative in their implementation approach [26–30]. Innovative organizational strategies attempted to optimize care delivery and improve quality for prenatal care, delivery [28–31] emergency obstetric care (EmOC) [32], newborn care [33] and infection control [34, 35]. Several approaches aimed to improve service processes and quality by providing management and leadership skills to health workers at various levels of the formal health system to empower them to identify and address challenges [36]. For example, in Egypt, health workers with management training implemented and evaluated quality improvement approaches, which were scaled-up after the study was completed [37]. Another innovative approach combined the organization of maternity service delivery with quality improvement aspects using a checklist for safe delivery practices, inspired from one previously utilized for intraoperative safety [38]. In Nepal, an effective quality of care model used for family planning was applied to EmOC which involved the setup of quality teams trained to evaluate quality of care on a monthly basis, develop and implement an action plan for quality improvement and remain accountable for progress through regular reviews [32].

Various innovative approaches were identified which relate to the delivery of facility-based mental health care [39], community- or family-based MNH nutrition and breastfeeding [40–51], kangaroo mother care (KMC) and prenatal care at both levels [52–65]. A study in South Africa incorporated mental health care for pregnant women into existing primary care services such as antenatal care visits and postnatal telephone follow-up [39]. Also in South Africa, facility-based KMC implementation has progressed through facilitated trainings, achievement of specific indicators outlined in an implementation tool as well as progress monitoring performed via in-depth interviews [40–51]. Implementation of KMC has been found challenging, and several RCTs on its use in low resource communities found no effect on mortality outcomes [52, 53].

Innovative nutritional approaches to improve maternal and newborn health include new micronutrient supplementation program strategies, involving zinc, iron, calcium or early prenatal food supplementation, and have been tested to improve antenatal nutrition and child health outcomes [40–46]. One pre-post study with control areas in villages in Egypt, for example, evaluated a positive deviance approach, basing an antenatal education and supplementation intervention on practices of positive outliers. It found that with this approach, women were more likely to report increased birth weights of their infants and higher food intake [45]. Finally, efforts to increase awareness and promotion of breastfeeding have involved the use of new, targeted promotion strategies, delivery systems and the mainstreaming of the practice in the scale-up of MNH programmes [47–51].

Most service delivery studies were observational in design and investigated care delivery outcomes, such as breastfeeding rates, satisfaction or knowledge scores. Overall, studies provided limited data on the effectiveness of health care delivery interventions on health outcomes.

### Medical products and health technologies

Innovative technology approaches and appropriate devices and medicines to promote MNH in resource-limited environments aim at improving service delivery through the supply-side. Many novel medical products and health technologies for safer births and improved newborn care are in development globally, but strategies to make them available in LMICs are unclear, and few have been implemented [66]. The insufficient development of distribution channels and lack of incentives for various stakeholders to test and disseminate products and technologies have been barriers to making them available at the point of care [67].

Peer-reviewed studies describing the effect of novel health technologies on health outcomes are limited in number and design. Several narrative reviews on maternal or newborn technologies are based on gray literature and provide limited analysis beyond descriptions of devices [66, 67]. Many MNH technology approaches are low-cost iterations of known devices based on simplified (low-tech) construction and production principles [68]. However, studies do not address criteria as to what makes these innovative approaches appropriate for LMICs.

The array of maternal health technologies include non-pneumatic anti-shock garments to stabilize and resuscitate hypovolemic shock in pregnant women, automated blood pressure devices tailored for low resource settings, single use obstetric emergency kits, and low-cost, low-tech devices such as portable obstetrical ultrasound equipment [38, 66]. Low-cost, low-tech birth simulators are available and have been used to train various cadres of providers in safe delivery techniques [38]. Partographs are an example of interventions aiming to increase the quality of care, which have long been in use and are now being adapted for further use in LMICs. A simplified partograph has been developed by WHO to monitor stages of delivery, and clinical RCTs conducted suggest it is useful in improving care [38]. Other innovative approaches aim at facilitating geographic access to care through low-cost transport options to EmOC facilities such as bicycles and motorbikes [68, 69].

Only a few studies provide clinical outcome data, such as those on non-pneumatic anti-shock garments suggesting that their use reduces observed blood loss and rates of hysterectomy [70, 71]. Clean birth kits have been suggested as an innovative approach, but evidence to support their impact on health outcomes is inconclusive, particularly in the community setting [72, 73]. A study from Bangladesh describes a balloon condom catheter to treat intractable uterine bleeding, but provides no clinical data [74]. Innovative use and storage of medicines for women include community-based administration of Misoprostol, simpler and safer Oxytocin delivery using the Uniject device and a foilized polyethylene pouch to store Nevirapine [74, 75]. Likewise, chlorhexidine is not a new intervention, but its innovative delivery and use for umbilical cord care in the first 24 h of life in LMIC have been shown to reduce neonatal nosocomial infections and mortality [76, 77].

A descriptive review on newborn health technology [67] suggests that there is increasing attention to low-cost, low-tech infant warmers [68], neonatal resuscitators [78], and phototherapy devices for the therapy of hyperbilirubinemia [79]. A variety of low-cost, low-tech pulse oximeters are in development; some are cell-phone based while others are marketed primarily for intraoperative patient safety purposes [80].

Although technologies and devices might need adaptations to meet needs in different countries, they are usually not developed with a certain region or country in mind. Few devices are being marketed and sold, with the exception of low-cost thermal devices [67] and several low-cost scales and temperature indicators distributed by NGOs [81].

### Health workforce

Innovative health workforce approaches address the shortage in human resources by enhancing their knowledge, skills, and competencies, while aiming at their retention in LMICs. Many innovative workforce approaches involve novel training programmes or approaches to improve the supply side of MNH and to expand the scope of existing health worker cadres. Various innovative workforce approaches address skilled workers, such as training of professional midwives in newborn care [82, 83] or providing additional training for medical doctors and other health workers in neonatal resuscitation using simulations [84]. Various international organizations have come together to form a network through which they have committed to train providers [85]. To facilitate the connection between trainers and trainees in settings where in-person trainings are difficult or impossible, innovative workforce education strategies uses electronic teaching (e-learning programmes) or continuing education through phone texts [86–88].

Creating and training new types of health workers, such as newborn aides in Neonatal Intensive Care Units [89], has shown promise in expanding aspects of coverage and quality. A review of randomized and non-randomized controlled studies that investigate strategies incorporating training and support of traditional birth attendants (TBAs) found significant reductions in perinatal and neonatal mortality [90]. A common approach is task shifting, the delegation of duties from more skilled medical personnel to non-physician or intermediate cadres of health workers. Other approaches directed at community health workers (CHWs) and TBAs assign them MNH responsibilities related to community-based antenatal, delivery and postnatal care. A similar strategy has also been used for the delivery of EmOC and anesthesia services [91–94].

The scope of obstetrics practice of mid-level health care providers (clinical officers) varies widely by country, but their performances for the tasks they are assigned are comparable to those of physicians. A meta-analysis of non-randomized studies found that clinical officers and doctors did not differ significantly in key outcomes for caesarean section and detected no significant differences in maternal or neonatal mortality for cesarean sections performed by the non-physician clinical officers versus medical doctors [91].

### Health financing

Innovative health financing models address limitations in access to quality care due to financial constraints, targeting care recipients or providers. Various innovative financial programmes aim at attenuating or reducing financial barriers to care, and at improving coverage and usage of MNH from supply, demand, or both sides.

Conditional cash transfers (CCT) provide financial assistance to low income families; subsidies are contingent upon various conditions such as meeting certain preventive health care requirements or school attendance [95]. CCT programmes identified in South Asia tend to be specifically focused on maternity care [96, 97], often removing MNH service user fees. This method can also improve demand for facility delivery as suggested in observational pilot studies conducted in Ghana, Senegal, and Bolivia [95, 98–104].

Voucher programmes are intended to directly provide funds for MNH services to target groups of mothers and newborns, particularly among low income or high-risk populations. Studies identified assessed programmes with vouchers distributed free or highly subsidized to eligible women to finance transport to a facility and to reimburse providers for delivery services. Other approaches aimed at lowering financial risks for households include community-based health or obstetric insurance (mainly in West Africa, South America, and China) [98, 105–109] and cash incentives for skilled facility delivery [110, 111].

Incentives for health workers aim to increase supply and quality of services through performance-based payments [112, 113] and full reimbursements of training and other costs [104]. Performance-based incentives are cash or other rewards and compensation to health workers for certain services. Some incentive programmes are contingent on provider performance outputs, referred to as results-based funding or paying for performance, such as the performance-based financing scheme in Rwanda [98].

Most published innovative financial approaches were developed, piloted, and implemented in South Asia. Some were scaled-up on a state level (India) [96] or national level (Rwanda, Nepal) [106, 110, 111]. All studies included in this review were retrospective and quasi-experimental in design, investigating mostly changes in health care supply or utilization, aside from an RCT of pay-for-performance in Rwanda, which found a significant increase (23 %) in the probability of a woman delivering in a facility [112].

### Community ownership and participation

Innovative approaches increasingly aim at strengthening community-based health mechanisms that improve links to and structures associated with primary health care. Innovative community ownership and participation approaches are complex, multifactorial interventions which often simultaneously address demand, supply, and quality aspects. Community-based intervention packages (CBIPs) are usually facilitated by women’s or mothers’ groups or by community-based health workers, often females [114]. Interventions consist of health promotion and disease prevention activities through care delivery and health education conducted in the communities or at the home during pregnancy, delivery and the postnatal period.

Various community-based MNH strategies involved women’s or mothers’ groups as channels of community ownership. For example, female facilitators in Makwanpur, Nepal convened monthly women’s group activities to improve perinatal health and supported groups through an action-learning cycle in which they identified local perinatal problems and formulated strategies to address them [115]. As a result, MMR was lower in intervention clusters with the women’s groups intervention than in control clusters, and women were more likely to have had antenatal care, institutional delivery, trained birth attendance, and clean delivery care [115]. Results from a cRCT in Sylhet (Bangladesh) indicated that a home care strategy to promote an integrated package of preventive and curative newborn care was effective in reducing NMR, whereas women’s group activities had no statistically significant effect on NMR [116].

Further community-based intervention packages have been evaluated in Hala, Pakistan [117, 118] and Mchinji District, Malawi [119]. These studies were large cRCTs including tens of thousands of mothers and newborns each assessing maternal and neonatal mortality outcomes, providing high-quality evidence in their respective local contexts. In a trial in Gadchiroli (India), home-based care and health education reduced the incidence of neonatal morbidities and low birth weight in the community and improved maternal knowledge and caretaking behaviors [114]. A community-based intervention in Shivgarh (Uttar Pradesh, India) focused on the provision of essential newborn care and on prevention of newborn hypothermia, and was associated with a reduction in the neonatal mortality rate (NMR) of about 50 % [120].

In contrast to large reductions in mortality reported in similar trials undertaken in Nepal and India, a large trial in Bangladesh evaluating participatory action and learning groups for women to develop and implement strategies to address MNH problems did not detect a statistically significant effect on NMR [121]. Contextual factors – including socio-cultural aspects and gender-based barriers – may influence participation in specific activities associated with community health. This study indicated that process-related factors as well as local context may have had a role in the intervention’s effectiveness. Specifically, poor conditions for transport and signs of gender-based barriers seemed to affect women’s access and participation in this setting. The Integrated Management of Neonatal and Childhood Illness (IMNCI) programme in Haryana (India) combined CHW postnatal home visits to treat or refer sick newborns with women’s group meetings [122]. A meta-analysis of data from studies showed that while community participatory approaches are not associated with improvements in MMR, they have been shown to reduce maternal morbidity, stillbirths and NMR, and improve referral to health facilities as well as breastfeeding rates [123].

As careseeking and referral are core components of community-based approaches, it is essential to make a strong linkage between the facility and community. Innovative strategies to foster this linkage include establishing an integrated package of care within the national system at community and facility levels, and creating a network of providers across all levels to enhance the capacity and quality of care [124–128]. At the centre of community-based approaches are ownership and participation, mobilized through community involvement and engagement, participatory learning activities and problem solving with local community and facility representatives, as well as positive deviance behavior change activities [129].

Most studies in this building block were conducted in rural settings. Most of the community-based approaches emphasize prevention and capacity building. The value of community participation and ownership is well established; however, besides mechanisms such as women’s groups and community-based approaches of follow-up with CHWs, there is little research published on other innovative mechanisms and approaches to facilitate community participation and mobilization in MNH.

### Leadership and governance

Innovative leadership and governance initiatives, related to the formation of partnerships and the formulation and implementation of national MNH policies, are considered part of the enabling environment for MNH and address supply, demand and quality. Studies discuss systems issues and historical developments, and describe efforts overarching the before mentioned areas in their political context.

Partnerships for MNH include public-private partnerships between governments and private obstetricians, nurses or midwives, international or regional partnerships and interagency task forces to enhance capacity and quality of MNH care. Several large-scale public-private partnerships were initiated in South Asia, where district health authorities, as care providers, purchased packages of services for the poor from the private sector. For example, a public-private partnerships in Gujarat, India connected 800 obstetric providers to provide health care to poor women and increased the proportion of women delivering at health facilities [94]. Additional innovations include health system-wide reforms; use of situational analyses, evidence and policies to develop implementation packages and national strategies; integrating skilled birth attendance into national policy; increasing political commitment; and creating rights-based programming and micro-planning strategies to increase access, coverage and quality of care.

The strategic use of global and national data and partnerships focused on newborn-related evidence for advocacy and planning is a MNH policy-making strategy that has influenced similar strategies in other countries [124]. Nepal was the first low-income country to create a data-driven national newborn strategy, the Community-Based Newborn Care Package [124, 130].

Most evidence on leadership and governance is documented in case studies and policy analyses, focusing on enabling factors of policy making. In a quasi-experimental setting in the Phillipines, a system reform for maternal health was shown to be associated with an increase in the rate of facility-based deliveries, as MMR declined more in reform areas than in comparison areas during the time period of gradual implementation [131]. Few observational policy evaluations included population data.

## Discussion

As indicated by the results of this review, the landscape of innovative MNH approaches is complex and diverse, and knowledge management is a challenge for country MNH programme planners and managers. This analysis’ results provide a geographical orientation of innovations and a summary of innovative MNH approaches within relevant health system building blocks. Our study aims to facilitate knowledge management and dissemination about innovative MNH approaches by providing an overiew of the landscape and related evidence. Thus, it allows researchers and innovators to identify gaps and to develop and appropriately evaluate new projects.

Most innovative MNH approaches identified in this review are adaptations and iterations of solutions from other settings. While the literature provides valuable lessons on development and implementation, evidence for the health outcomes and impact of such innovations in LMICs is often limited. Innovative approaches address various barriers to health care access in LMICs, often simultaneously: geographic access, availability, affordability, acceptability, and quality [132]. Some interventions target specifically mothers, while others address both mothers and newborns, in a combination of elements across the continuum of care. A continuum of care approach is supported by empirical evidence suggesting positive, synergistic effects of strategies emphasizing interconnected care of mothers and neonates [133].

### Innovative MNH approaches in every health system block

In LMICs, delivery of effective quality care is challenging due to many barriers, which often relate to other health system building blocks. Barriers to **health service delivery** in resource-constrained settings include lack of or inadequate facilities and infrastructure including drug and equipment supply, distribution of health facilities inconsistent with population needs, insufficiencies in health worker training and continuous education, lack of incentives or other mechanisms for quality care and health worker retention, and insufficient standardization of care delivery. Studies of innovation in health service delivery usually assess improvements in service coverage [134]. Innovative packages often integrate multiple MNH interventions (e.g. training in EmOC, placement of care providers, refurbishment of existing health facility, infrastructure and improved supply of drugs and consumables and equipment for obstetric care), both at the district and sub-district level [135]. These are observational studies reporting performance or utilization outcomes, or concurrent changes in maternal mortality rate (MMR). A small cRCT assessed care utilization and client satisfaction [136].

Improving access to and availability of health services, including emergency services, are key strategies towards achieving the health-related MDGs, but innovative approaches also need to address quality and equity. Conclusions about the applicability and appropriateness of innovative approaches in LMICs are limited by the relative scarcety of controlled studies in the current published literature. While many interventions are specifically designed to improve the quality of care, most lack evidence on quality improvement of service delivery; few evaluations of quality improvement programmes assessed indicators of quality. Quality of care is not uniquely defined in MNH generally [137]. This lack of a definition of quality was also observed in studies on innovative approaches. Few studies investigated measures related to safety, effectiveness, efficiency, patient-centeredness, timeliness, and equity. Some papers on quality improvement projects did not report data on quality of care at all. Given the observational study design, causality of the observed effects cannot be attributed to the programme activities.

Most studies of the effects of innovative approaches in MNH care delivery are observational and do not account for potential confounders. EmOC evaluation studies measured mostly programme outputs using process indicators such as facilities per population [138], outputs such as the proportion of cesarean sections among deliveries [139], or outcome indicators such as met need for EmOC services [139–141]. Few studies investigated effects of EmOC implementation or improvement interventions on mortality [142], and mortality was often measured as procedure-related case fatality rate rather than population-based MMR [143].

More rigorous, applied research would add valuable evidence on the effectiveness, replicability, and scale-up of current innovations. Future studies on innovative health service delivery approaches should investigate measurable health and social impact. For equitable programming, outputs of care delivery and process outcomes need to be measured, as well as indicators of quality of care, of morbidity and mortality (including among vulnerable populations), and cost-effectiveness.

**Medical products and health technologies** for use in LMIC have received considerable attention recently, including from United Nations organizations [78, 144]. This review’s findings however point out that - with the exception of smaller pilots on anti-shock garments or clean delivery kits - the current body of published literature lacks rigorous evidence on safety, effectiveness, and potential side effects. Studies describe novel MNH technologies, but data on effectiveness, safety, and unintended effects are missing. Few technologies have been scaled up to mass production, which might lower the retail price and incentivize a supply chain to get appropriate and affordable technologies to mothers and newborns. Criteria for what makes technology appropriate for LMICs are unclear, as are issues of affordablity for end users in LMICs, and efficient strategies to make health technologies suitable and acceptable. While production costs or end user prices of technologies are not specified in the papers reviewed, even scaled-down high-tech versions are often too expensive for use in LMICs, and will require user training and device maintenance.

Safety and potential adverse effects need scrutiny in future studies. For example, women on antiretroviral therapy do not need breast shields to prevent transmission of infection to their children, and it is conceivable that these devices interfere with a mother’s ability to consistently breastfeed as recommended by WHO [145]. Most importantly, while there is a promising pipeline of innovative technologies in development, these need sustainable distribution strategies to reach mothers and newborns in resource-limited settings. Future clinical trials need to measure not only health outcomes, but also implementation, acceptability, and usage aspects to assess adverse effects on currently recommended best practices, in order to mitigate these risks and potentially reverse them.

The scarcity of skilled providers represents one of the main obstacles to the expansion of MNH care, especially for basic and comprehensive emergency obstretric care [146]. Many innovative **health workforce approaches** concentrate on task shifting and training. While the body of evidence is of varying rigor, evaluations indicate that these approaches might help narrow MNH delivery gaps due to the shortage in health workforce. While most governments favor approaches encouraging deliveries at health facilities, many health systems are still far from being able to offer comprehensive, skilled-birth attendance to all pregnant women. In the absence of sufficient capacity in human resources for skilled birth attendance, there is strong evidence from cRCTs and non-randomized controlled studies that strategies incorporating training and support of TBAs reduce perinatal and neonatal deaths [90]. Most studies on innovative health workforce approaches included in this review used indicators such as short-term knowledge retention or skills scores as outcomes. Few evaluated health outcomes, or longer-term retention of knowledge and development of skills.

In many LMICs, households bear most of the financial burden of MNH care. Families who are unable to pay out-of-pocket fees experience delays or even insurmountable barriers to accessing care, which can result in fatal outcomes and catastrophic household expenditures [101]. Recent **innovative health financing models** removing or reducing MNH service user fees (e.g. fee exemption for maternity care) have been shown to have positive effects on supply of and demand for MNH. Usually equity-driven, these programmes are designed to target the poor to subsidize cost or exempt fees for specific services, such as cesarean deliveries. Performance-based financing including pay-for-performance, performance-based incentives, results-based financing, or CCTs have increased access and utilization of MNH services [147]. These approaches are often part of more complex initiatives to improve MNH service delivery, and their specific effects on health worker performance, or on MNH indicators, is often difficult to discern [148]. There are few controlled evaluations of innovative financial approaches. Included studies focus on utilization outcomes, and few assess equity-focused targeting (i.e. how financial innovations meet the most vulnerable populations or improve MNH care among the poor). In the few observational studies that include a control area or group for comparison, the allocation of programme versus control was often unclear, and there was no or insufficient adjustment for potential confounders in the between-group analyses. Several studies included analyses of low-income subgroups to assess the equity effects of targeting as part of vouchers programmes [100, 149, 150]; positive equity effects have been inferred from interventions predominantly used by economically disadvantaged groups [151]. Study findings suggest that demand-side financing projects can be an effective way of reducing inequities in institutional deliveries, but an equity gap remains [103].

A rapidly increasing body of evidence from large RCTs **on innovative community ownership and participation** suggests that community-based care has positive effects on maternal and neonatal mortality. Innovative participatory approaches involve engagement of community leaders, behavior change activities, community health education, organization of community transport mechanisms, community-based packages of MNH care, and other forms of community participation and mobilization [129]. Community ownership and participation strategies can be innovative in their application of practical, culturally adapted processes, which build capacity for communities to develop and scale-up their own solutions [129]. By taking ownership and building on existing structures [15], community members can increase responsiveness to the health needs of the community and adopt behaviors that promote and preserve health [16]. These health effects increase with higher participation and population coverage [152]. The mechanism of how mothers’ and women’s groups achieve these effects are less clear [153].

Overall, the strong evidence confirmed in this analysis suggests that community-based interventions are an integral building block of a health system delivering effective MNH care, and that the aspects of community ownership and participation need particular attention.

**Innovative leadership and governance initiatives** at national and decentralized levels are essential to influence action on key health determinants and access to health services, and to ensure accountability [15]. These were based on partnerships and political coalitions at various levels to catalyze innovative approaches in MNH care. Political and strategic leadership and governance in resource-limited settings face tremendous challenges in a complex landscape. In the studies on leadership and governance, it is difficult to directly link policy changes to observed population-level outcomes, or to control results for environmental confounders. However, there is increasing recognition of the importance and commitment for the implementation of innovative MNH approaches [154]. Funding is limited, and so is the availability of reliable data and in many instances political commitment for women and children [155]. Innovative approaches in MNH will need concrete political and financial investments in high-yield and cost-effective interventions for approaches to succeed. Fueled with the funding and political support required to develop and implement innovative MNH approaches, our findings suggest that international political partnerships might be a decisive facilitating factor in the MNH care landscape.

## Limitations

This study aimed at providing an overview over the landscape of existing, published innovations in MNH. Given the study’s broad scope, it has has several limitations. First, the review only includes published studies, and not all studies might have been found during database searches. We therefore set our search terms very broad. Secondly, given the very nature of our scoping review, we did not overlook all existing innovations at the onset of our search. Due to the varied nature of interventions and study types included in the review, we could not undertake a metaanalysis of existing evidence. Third, we included studies with imperfect study designs and limited or missing outcome data. This allowed us to map a broader range of evidence, including indications where it is missing, and we rated lower grades of evidence accordingly. Finally, considerable time has elapsed from database review to publication. This means that while our study can give indications of the MNH innovations landscape and existing evidence and gaps, it should be considered potentially incomplete and interpreted with caution.

## Conclusions

Innovative approaches are key to improving equity in MNH services delivery. Our study suggests that important evidence gaps remain. Overall, very few studies assessed intervention effects on health outcomes. Rigorous randomized trials to assess interventions are rare; most evaluations are smaller pilot studies. Countries with the most progress in MNH did so by reaching out to the poorest and most remote populations, thereby improving equity in MNH service coverage. Measuring and documenting equity in evaluation studies is important to measure the potential of innovations to improve health equityand to identify and target population groups at higher risk of service inequity. Few studies focus on equity in health care delivery, which is necessary to ensure that quality care is available to all. Inequities in access, use, and outcomes of health care can be detected in subgroup analyses, comparing data of disadvantaged populations with national or regional data. We found few data on vulnerable subgroups, which limits an equity assessment for innovative health service delivery approaches.

Furthermore, in order for any innovative intervention to be scaled up in low-resource settings, evaluation studies need to consider cost, feasibility, and acceptability. The process of innovation does not end with implementation. Ultimately, innovative MNH approaches are only successful if they are sustainable and integrated into the health system. Innovative approaches in MNH care also will require innovative strategies for their evaluation. This will allow programme and policy planners to assess the potential of interventions and ultimately determine which approaches may work, and why.

## Additional file

Additional file 1: Figure S1. Flow chart study selection for the analysis. (DOCX 52 kb) Additional file 2: Table S1. Innovative approaches to maternal and newborn health care (by WHO Health Systems Building Block), related evidence, and implications for programming and implementation research. (DOCX 332 kb)
