# Supplementary material for: Innovative approaches for improving maternal and newborn health - A landscape analysis
Source: BMC Pregnancy Childbirth. 2015 Dec 17;15:337. doi: 10.1186/s12884-015-0784-9 (PMC4683742; doi:10.1186/s12884-015-0784-9)
Supplement: Additional file 1: Figure S1. — Flow chart study selection for the analysis. (DOCX 52 kb) [file 12884_2015_784_MOESM1_ESM.docx]

Figure S1 Flow chart study selection for the analysis

**Identification**

**9,413**

Citations identified and retrieved

**8,932**

Studies excluded by title as not relevant

**-51**

Multiple innovations in one paper (8)

Same innovation in multiple papers (13)

→ removal of duplicates

**12**

Studies included from hand search of references and authors’ collections

**59**

Studies excluded not providing information on MNH innovations upon full review

**208**

Studies included
in systematic analysis

**Inclusion**

**Screening**

**259**

Studies included in inventory

**Eligibility**

**481**

Abstracts screened

**306**

Full text article obtained for detailed evaluation

Annex B Study Protocol: Appraisal and grading of study quality for studies fitting inclusion criteria

We developed a grading system covering all types of included studies and reviews (see Annex 2 for grading decision tree). For quantitative study designs, we assessed study quality following the SIGN Grading System 13, and graded studies from 1 to 4, according to scientific rigor of the study design. Quality assessment criteria included study design, potential for selection bias, confounder, blinding, data collection methods, withdrawal/dropouts, and integrity of intervention. Study designs included systematic reviews, randomized controlled trials (RCTs), cluster randomized controlled trials (cRCTs), , quasi-randomized trials, controlled and uncontrolled pre and post studies, and controlled and uncontrolled interrupted time series (cITS and ITS) studies. We rated uncontrolled studies accordingly for study design and bias. We categorized non-analytic articles, case studies without outcome or output data, and expert opinion articles as grade 4. For qualitative study designs, we graded included studies as A, B, or C, according to their adherence to all, most, or few criteria for qualitative research, following recent standards on the reporting of qualitative research in mixed studies reviews (15-17).

Annex B: Study Protocol - Review Quality Assessment Guide (after SIGN)

For **systematic review/meta-analysis**:

1) Does the study address an appropriate and clearly focused question?

2) Is a description of the methodology included?

3) Is the literature search is sufficiently rigorous to identify all the relevant studies?

4) Is the study quality assessed and taken into account?

5) Are there enough similarities between the studies selected to make combining them reasonable?

**If review includes RCT:
GRADE 1++** All or most of the criteria have been fulfilled, and conclusions of the review very unlikely to alter.

**GRADE 1+** Some of the criteria have been fulfilled, low risk of bias, study conclusions unlikely to alter.

**GRADE 1-** Few or no criteria fulfilled, high risk of bias, study conclusions likely to alter.

**If review includes only case-control or cohort studies: GRADE 2+**

If paper compares intervention with control NO → Descriptive study

↓ YES Case study, series: **GRADE 3** Expert opinion: **GRADE 4**

Investigator assigned intervention/control NO → Observational study

↓

More than one group studied? NO →

↓YES

Measured at one time point? YES→

↓ NO

← YES Groups defined by outcome? NO →

↓ YES

Interrupted time series
 **GRADE 3**

Prospective controlled trial

- see over

Case control study

• Clear study question.

• Cases and controls from comparable populations

• Same exclusion criteria for both cases and controls

• Comparable percentage of each participated in the study

• Comparison between participants and non-participants similar

• Cases are clearly defined and differentiated from controls

• Exposure status measured in a standard, valid, reliable way

• Potential confounders identified and accounted for

• Confidence intervals provided

Cross sectional study
 **GRADE 3**

**Grading for cohort or case-control studies**

**GRADE 2++** All or most of the criteria have been fulfilled, high quality study, very low risk of bias, highly probable causal relationship

**GRADE 2+** Some of the criteria have been fulfilled, good study, low risk of bias, moderatly probable causal relationship

**GRADE 2-** Few or no criteria fulfilled, high risk of bias

Cohort study

• Clear study question.

• Comparable groups.

• Number of people screened for inclusion in each arm.

• Indicates percentage of drop- outs in each arm.

• Outcomes clearly defined.

• Assessment of outcome blind to exposure status.

• Measure of assessment of exposure reliable and valid.

• Potential confounders identified and accounted for

Intervention assigned randomly

↓ YES → NO ↓

Non-randomized controlled trial

Quasi-experimental studies

1. Are study question, population, intervention/control, outcomes clearly described?
2. All relevant outcomes measured in a standard, valid and reliable way?
3. Was design/analysis adjusted for confounders?
4. Were drop-out accounted for and analysis intention to treat?
5. Results are comparable for all sites (if multi-site)?

**GRADE 1+** Most criteria have been fulfilled. Those criteria that have not been fulfilled or not adequately described are thought unlikely to alter the conclusions.

**GRADE 1-** Few or no criteria fulfilled. The conclusions of the study are thought likely to alter.

(Cluster) RCT

1. Are study question, population, intervention/control, outcomes clearly described?
2. Was an adequate concealment method used?
3. Were subjects and investigators kept ‘blind’ about treatment allocation?
4. Were intervention and control groups similar at start and only difference between groups the treatment under investigation?
5. All relevant outcomes measured in a standard, valid and reliable way?
6. Was design/analysis adjusted for confounders?
7. Were drop-out accounted for and analysis intention to treat?
8. Results are comparable for all sites (if multi-site)?

**GRADE 1++** All or most of the criteria fulfilled, conclusions of the study very unlikely to alter.

**GRADE 1+** Some of the criteria fulfilled, those not adequately described unlikely to alter the conclusions.

**GRADE 1-** Few or no criteria fulfilled. The conclusions of the study are thought likely to alter.

Qualitative study

1: Clearly formulated objective and question (relevant to MNH innovation)

2: Qualitative approach or design or method appropriate

3: Description of context

4: Description of participants: How were the setting and the subjects selected, and were they information-rich?

5: Discussion of researcher’s reflexivity: What was the researcher's perspective, been taken into account?

6: Description of qualitative data collection and analysis: Methods for collecting data described in enough detail? Quality control measures implemented?

7: Conclusions drawn and justified by results? Results credible and relevant? Findings of the study transferable?

**A:** All criteria fulfilled, rigorous qualitative study

**B:** Criteria mostly fulfilled, good qualitative study

**C:** Criteria poorly fulfilled, qualitative study of limited rigor
