# Supplementary material for: Innovative approaches for improving maternal and newborn health - A landscape analysis
Source: BMC Pregnancy Childbirth. 2015 Dec 17;15:337. doi: 10.1186/s12884-015-0784-9 (PMC4683742; doi:10.1186/s12884-015-0784-9)
Supplement: Additional file 2: Table S1. — Innovative approaches to maternal and newborn health care (by WHO Health Systems Building Block), related evidence, and implications for programming and implementation research. (DOCX 332 kb) [file 12884_2015_784_MOESM2_ESM.docx]

**Table S1 Health Services**

| **Innovative MNH approach** | **Summary of Evidence** | | | | **Implications** |  |
| --- | --- | --- | --- | --- | --- | --- |
|  | **Evidence** | **Type of Study** | **Reference** | **Grade** | **Programming** | **Implementation Research** |
| **Quality Improvement** | | | | | | |
| Management and leadership (M&L) development for MNH services quality improvement | After M&L skills development, case fatality rates for pre-eclampsia decreased from 3.1% to 1.1%, hemorrhage from 14.8% to 1.9%, stillbirths reduced by 36%, MMR reduced by 34% (decreased from 496 to 328 per 100 000) in Ghana. | Interrupted time series | ^1^ | 3 | Management and leadership development activities could be implemented at various levels of health care systems; shown potential for scale-up in Egypt | Assessing if leadership interventions have impact on health outcomes. |
|  | After M&L skills development, overall MMR and RR of maternal mortality was lower than historical comparison in Nigeria; yearly increase in live births. Less presentation–intervention intervals over 30 min among cases of maternal mortality. | Cross-sectional | ^2^ | 3 |  |  |
|  | A pilot programme of M&L development scaled up to 184 health care facilities, family planning visits and number of prenatal increased; at same time, MMR in region reduced from 85.0 to 35.5 per 100,000 live births | Cross-sectional | ^3^ | 3 |  |  |
| WHO Safe Childbirth Checklist, an innovative, evidence-based standardized protocol for MNH care | A checklist verifying use of 29 essential practices to prevent childbirth-related mortality evaluated in India. With the checklist, practices increased from 10-29 to 25-29, improvement in the delivery of 28 out of 29 individual practices. | Pre post | ^4^ | 3 | Checklist needs to be adopted for local circumstances; WHO group offers to give guidance in that process. | Scale-up study including health outcomes currently being conducted |
| Implementation of redesigned protocols of MNH care following quality improvement | Women's satisfaction levels improved after implementation of redesigned care processes, caesarean birth decreased (30% compared with 42% previously) in a study in Iran. | Interrupted time series | ^5^ | 3 | QI intervention was based on women's needs assessments and clinical recommendations | Testing whether model might be adopted to improve compliance with evidence-based guidelines elsewhere |
| Collaborative quality improvement of a network of sites that work together for a specified period of time to rapidly achieve significant improvements in a focused topic through shared learning | QI models for MNCH include COPE (Client-Oriented, Provider-Efficient Services), Fully Functional Service Delivery Point (FFSDP), HIVQUAL, Improvement Collaborative, Improving Newborn Health, Partnership Defined Quality (PDQ), Private Sector Quality Improvement Package, Quality Design/Redesign, Reaching Every District (RED), and Standards-Based Management and Recognition  (SBM-R) | Report | ^6^ | 4 | QI models can provide programmatic guidance and be applied at all levels of the health system, using existing resources, to put in place long-lasting  changes in how care is provided | Assessing in evaluation studies beyond case studies |
| Comprehensive intervention packages based on QI approaches | Intervention package with 1) establishment and certification of comprehensive emergency obstetric and newborn-care centres 2) continuous delivery services at the primary health centre level, 3) incentives for medical officers in Tamil Nadu, India | Case study | ^7^ | 3 | QI activities need to be monitored and sustained | Assessing in continuous evaluation studies |
|  | With intervention package in Ningxia, China incl 1) guidelines for providers and mothers, 2) incentive plan, 3) public health promotion, 4) training of healthcare providers , 5) maternity waiting homes, and 6) upgrading critical equipment and facilities, MMR decreased, fewer children were born at home, women with prenatal care and prenatal visits in the first trimester increased | Interrupted time series | ^8^ | 3 |  |  |
|  | QI led to development of a comprehensive postnatal package of care, with three postnatal assessments by providers in maternity and maternal and child health clinics in Kenya; the QI process improved counseling scores for danger signs in the newborn, infant feeding, quality of care index for newborn. | Pre post | ^9^ | 3 |  |  |
| UNICEF Safe Motherhood programme | Implementation of the programme increased rate of prenatal visits in the first trimester (from 38.9% to 76.1%), overall prenatal visits (82.6% to 98.3%), women mobilized to deliver in hospitals (62.7% to 94.5%), hospital delivery rates (31.1% to 87.3%); MMR reduced 34.9% from 91.76 to 59.74 per 100,000 live births. | Pre post | ^10^ | 3 | Endorsed by both Chinese Ministry of Health and UNICEF | Sustainability needs to be assessed in continuous evaluation studies; needs to test neonatal outcomes |
| Special Care Newborn Units to provide quality level II newborn care services in remotest districts in India | Human resources, equipment and other constraints are challenges in scaling up specialized NICU units in remote districts in India. | Case study | ^11^ | 4 | Very limited evidence on implementation | Assessing in qualitative and quantitative evaluation |
| Infection control programme in reducing nosocomial infections in neonatal unit | In Senegal, rate of nosocomial bloodstream infections decreased from 8.8% to 2.0%, rate of infections/patient-day decreased from 10.9 to 2.9/1000 patient-days; overall mortality rates did not differ significantly; neonates on antimicrobial therapy decreased from 100% to 51% of at-risk infants; incidence of drug resistant bacteria decreased | Interrupted time series | ^12^ | 3 | Simple, low-cost and sustainable interventions shown in various settings to control nosocomial infections | Sustainability and scale-up in other settings to be evaluated. |
|  | In a programme in Bangladesh, declines in episodes of suspected sepsis (47%), cases of culture-proven (61%) sepsis, patients with a clinical diagnosis of sepsis (79%), and deaths with clinical (82%) or cultureproven sepsis (50%) | Pre post | ^13^ | 3 |  |  |
| Package of MNH interventions at institutional level [Programa de Atenção Integral à Saúde da Mulher (PAISM)] | There was good adherence to a protocol of maternal care in Brazil (two home visits and consultation of maternity cards or patient records during prenatal and hospital care). Only 38.6% of all women enrolled in the programme received "adequate" care according to protocol. | Cross-sectional | ^14^ | 3 | Such programmes need strategies for early implementation and prenatal care initiation before the end of the first trimester. | Limited data available on process and health outcomes. |
| Mental health care to pregnant women using existing resources in primary care [Perinatal Mental Health Project (PMHP)] | In a programme in South Africa, 90% of all women attending antenatal care were offered mental health screening with 95% uptake, 32% qualified for referral to counseling | Case study | ^15^ | 3 | This apporach used a collaborative, step-wise manner to support mental health in pregnant women within existing resources in primary care. | Outcomes evaluation to assess effectiveness and potential for scale-up |
| Provision of basic and emergency obstetric equipment and training to facilities, community education on maternal health | The ‘‘Reducing and Eliminating’’ EmOC intervention, scaled-up in China to 1,000 counties, compared to historical data, decreased MMR by 50%, increased hospital delivery rate. | Interrupted time series | ^16^ | 3 | The Chinese government used proven interventions and targeted rural areas with economic development and a high burden of maternal death. | Continuous evaluation of scale-up activities useful, and control areas adjusted for economic development. |
| Application of quality of care model from family planning to EmOC | Behavior change communication, human resource capacity building, strengthening of infrastructure implemented at 3 district hospitals in Nepal improved the understanding of quality improvement at local and national levels. | Case study | ^17^ | 4 | The program developed and implemented a quality of care approach at three district hospitals in Nepal . | Needs evaluation study based on Nepalese quality of care evaluation framework for maternity services. |
| **Skin-to-skin Care** | | | | | | |
| Community-based KMC | This study in Bangladesh showed no effect of community-based KMC (CKMC ) on NMR. | RCT | ^18^ | 1+ | Many newborns are not weighed at birth, making the diagnosis of prematurity problematic. | Need to assess why implementation was weak, solve problem of extensive missing birth weight |
|  | No effect of CKMC in the same trial in Bangladesh on outcome and impact measures of newborns held skin-to-skin less than 7 hrsd/day. | RCT | ^19^ | 1+ | Most women who were taught CKMC hold their newborns skin-to-skin, but do so in a token manner unlikely to improve health or survival. | Effective training and postpartum support need to achieve adequate skin-to-skin practices, before scaling up and further evaluation are done. |
| Kangaroo mother care (KMC) implementation tool | The paper proposes the validation of a monitoring model for KMC. | Qualitative study | ^20^ | C | Might facilitate implementation and evaluation of KMC programmes. | Qual. validation study conducted, but with unclear methods. Needs validation. |
|  | Monitoring model for KMC evaluated qualitatively. | Qualitative study | ^21^ | C | Paper presents main issues in the establishment of kangaroo mother care | Needs validation. |
|  | Tool used to evaluate KMC provided in hospitals, with support from regional steering committee | Case study | ^22^ | 3 | Hospital friendly hospitals score higher in KMC performance. | Needs validation of instrument and evaluation of programme. |
| Kangaroo mother care implemented in government hospitals | In Minas Gerais in Brazil, the method is being implemented effectively in government-run hospitals. Periodic re-training is needed. | Qualitative study | ^23^ | B | Limited programmatic insight from study. | Needs validation. |
| KMC implemented at hospital with facilitation | Successful implementation in 34 hospitals in KwaZulu-Natal Province, South Africa, was achieved in most of the hospitals irrespective of the strategy used. Facilitation improved implementation assessed per “progress score”. | RCT | ^24^ | 1- | Some sites do not need facilitation for successful implementation. | Needs evaluation of optimal facilitation strategy. |
|  | In Gauteng, South Africa, a study found no difference in kangaroo mother care implementation with face-to-face facilitation at hospitals vs education at a workshop off-site. | RCT | ^25^ | 1- | The choice of outreach strategy should be guided by local circumstances, cost and the availability of skilled facilitators. |  |
| **MNH nutrition** | | | | | | |
| Zinc as adjunct treatment for antibiotic regimen in newborns treated at urban hospitals | Additional treatment with zinc led to fewer treatment failures in a trial in New Delhi, India, and the disease-specific CFR was lower with zinc adjunct treatment. | RCT | ^26^ |  | Zinc could be given as adjunct treatment to reduce the risk of treatment failure in young infants with probable serious bacterial infection. | Further evaluations underway on other applications of zinc. |
| Iron fortification of wheat and corn flour to prevent anemia in pregnancy | Women at a prenatal clinic in Brazil had higher Hb levels and less anemia after the introduction of flour fortification | Pre post | ^27^ | 3 | Limited inferences on programming possible based on this study. | Prospective evaluations warranted. |
| Early initiation of prenatal maternal food supplementation | In Bangladesh, early initiation of prenatal food supplementation was associated with less food-insecurity and more maternal-infant interaction | RCT | ^28^ | 1- | More food insecure families have a lower quality of maternal-infant interaction. | Prospective evaluations warranted. |
| Calcium supplementation in pregnancy | In the Gambia, calcium supplementation from 20 weeks gestation to delivery did not lead to differences in breast milk concentration or infant measures | RCT | ^29^ | 1- | No effectiveness shown. | No effectiveness shown. |
| Multiple micronutrient supplementations in combination with early initiation | In Bangladesh, there was no difference in hemoglobin concentrations with various micronutrient schemes, i.e., early vs usual initiation group. Early initiation of multiple micronutrients had a lower mortality rate than usual initiation or iron and folic acid alone. | RCT | ^30^ | 1++ | Among pregnant women in poor communities in Bangladesh, treatment with multiple micronutrients resulted in decreased childhood mortality. | Strong evidence from single trial, and scale-up in South Asia and elsewhere needs to be assessed. |
| Positive deviance approach to improve antenatal nutrition | In villages in Egypt targeted with an antenatal education and supplementation intervention, women were more likely to report increased birth weights of their infants, more likely to report higher food intake. | Pre post with control area | ^31^ | 3 | The Government of Egypt and partners are scaling up the elements of the project. | Might be evaluated in other settings. |
| Integrated food supplementation programme for pregnant women | In a program in Bangladesh, only a third of eligible malnourished women received supplementation correctly; supplementation in this study did not show effect on maternal weight gain or neonatal birth weight/LBW rate. | Cross sectional | ^32^ | 3 | The Bangladesh Integrated Nutrition Program (BINP) might not have targeted optimally. | Needs appropriately powered evaluations stratified by targeted populations. |
| **Nutrition: Breastfeeding** | | | | | | |
| Breastfeeding "gear model" to promote implementation of breastfeeding promotion interventions | Innovative aspects of breastfeeding include communication and mass media campaigns, innovative facility- and community-based delivery systems (baby-friendly hospitals, peer facilitators, etc.) | Mixed-methods review | ^33^ | 4 | Model aims to inform breastfeeding programming generically. | Validation needed to test the application of the model for programming. |
| Post natal home visits to improve breast feeding and postnatal morbidities | In Syria, mothers who were visited at least once by a midwife for postpartum care were more likely to breastfeed. No differences in other outcomes. | RCT | ^34^ | 1- | Limited programmatic inferences from this study. | Appropriately powered trials necessary to assess health impacts. |
| Peer-counseling to promote breast-feeding | Peer supervision creates the highest cost in peer-counseling to promote breast-feeding in a project in Uganda. Total project costs were US$139 per mother and US$ 26 per visit. | Costing study | ^35^ | 3 | Not an efficacy trial. | Effectiveness of this intervention needs to be tested. |
| Community volunteers to promote exclusive breastfeeding | In Sokoto, Nigeria, training community volunteers in breastfeeding promotion increased intention to exclusively breastfeed. All infants of mothers who had received counseling did breastfeed. | Pre post | ^36^ | 3 | Baseline data suggests that many mother are still not exclusively breastfeeding. | Evaluation necessary to assess intervention’s health impacts. |
| Mainstreaming exclusive breastfeeding into maternal and child health programmes for scale-up | Key processes for the scale-up of exclusive breastfeeding include the creation of evidence-based policies and guidelines, and implementation strategies and plans for all strata of society. | Literature review | ^37^ | 4 | Breastfeeding remains single most important preventive intervention against child mortality. | Programme and policy evaluations needed. |
| **Prenatal Care** | | | | | | |
| Maternity waiting homes combined with MCH services and micro credit / income generation activities | Ethnic minorities encounter significant barriers to using waiting homes. In Lao, micro-credit and income generating activities to co-exist with medical protocols. | Qualitative study | ^38^ | C | Waiting homes couples with approaches from other building blocks | Programme and policy evaluations needed. |
| Maternity waiting homes combined with MCH services | Most facility-based deliveries were among women within 5 km; waiting homes in rural Timor-Leste did not improve institutional deliveries among women living more remote. | Pre post | ^39^ | 3 | Programming might need to address other barriers | Might need evaluation of regional barriers to care. |
| Newly implemented maternity waiting homes | In Nicaragua, both pregnant women as well as their husbands need to be informed about waiting homes, and women need be connected to postnatal care. | Mixed methods study | ^40^ | 4 | Study on its formative stages | Programme and policy evaluations needed. |
| Yoga in high-risk pregnancy | In Bangalore, India, yoga offered to women with high-risk pregnancy lowered rates of pregnancy induced hypertension, preeclampsia, diabetes, growth retardation, small-for-gestational age neonates, and those with low APGAR scores | RCT | ^41^ | 1- | Has been practiced for ages, but use to prevent high-risk complications is innovative. | Needs evaluation of effectiveness in other target populations. |
| Interpersonal-psychotherapy-oriented childbirth education programme for first-time childbearing women | In China, women in intervention groups scored higher for level of social support, maternal role competence, had less postpartum depressive symptoms, better psychological well-being | RCT | ^42^ | 1- | Integration into routine care needs ongoing evaluation. | Needs large studies with rigorous methodologies, potentially enrolling multiparous women |
| Group prenatal care to take account of women's views and specifically address their need for information, support and communication | Significant improvement in client satisfaction and prenatal care utilization with group care in Iran; women in group care are more likely to have adequate ANC | cRCT | ^43^ | 1- | Group prenatal care might address lack of peer support, cultural and traditional practices, where low-quality health services interfere with implementation of prenatal care. | Needs large studies with rigorous methodologies |

**B. Health Technology**

| **Innovative** **MNH approach** | **Summary of Evidence** | | | | **Implications** |  |
| --- | --- | --- | --- | --- | --- | --- |
|  | **Evidence** | **Type of Study** | **Reference** | **Grade** | **Programming** | **Implementation Research** |
| **Maternal technologies** | | | | | | |
| Non-pneumatic Anti-Shock Garment to stabilize and resuscitate hypovolemic shock | After device implementation, observed blood loss was 50% lower, rates of hysterectomy declined (8.9% vs. 4%), and CFR decreased from 8.5% to 2.3% in Egypt, Nigeria | Pre post | ^44^ | 3 | Device can be used by nurses; needs to be tested at community and household levels | Needs prospective, controlled study, cost-effectiveness assessment |
|  | Blood loss, time to recovery from shock improved after introduction of the device compared to time period before. | Pre post | ^45^ | 3 |  |  |
|  | Technology description | Narrative review | ^46^ | 4 |  |  |
| Automated BP devices suitable for low-resource settings | Devices distributed to 11 clinics and re-assessed 5 times over 36 months. Devices were used frequently with high levels of user satisfaction and good durability in primary health-care facilities in rural Tanzania. | Interrupted time series on acceptance | ^47^ | 3 | Pricing, distribution, and maintenance unclear; hardly useful without therapeutic modalities in place | Needs testing with variety of users, in settings assessing therapeutic consequences |
| Single-use obstetric emergency medical kits | After the introduction of birthing kits, facility-MMR decreased from 389 to 234 per 100 000 live births pre/post, 30% reduction, non-significant ( P=0.09) | Pre post | ^48^ | 3 | Primary use for obstetric emergencies in resource-poor setting | Needs prospective, controlled study, CE assessment |
| Misoprostol for community-based use | Technology description, no data | Narrative review | ^46^ | 4 | Limited experience with community-based application | Need to test use among various health worker cadres |
| Storage and application system for oxytocin delivery | Technology description as tested in Indonesia | Narrative review | ^46^ | 4 | Being tested and scaled up by US based NGO; clinical trials conducted on acceptability | System can also be used for other drugs |
| Balloon condom catheter to treat intractable uterine bleeding | Technology description as tested in Bangladesh | Narrative review | ^46^ | 4 | Few clinical testing available beyond proof of concept | Needs testing for effectiveness, efficacy, appropriateness for various health worker cadres |
| Foilized Polyethylene Pouch to store Neviparine | Technology description, no data | Narrative review | ^49^ | 4 | Few clinical testing available beyond levels of preservation | Might be considered for PMTCT programme logistics |
| Low-cost, portable OB ultrasound and Doppler | Technology description, no data | Narrative reviews | ^50^ | 4 | Devices in development, no pricing | No published clinical trials yet |
| Simplified partograph | Simplified partograph developed by WHO shown to be more user-friendly (P=0.002) and more likely to be completed; associated with fewer cesarean deliveries and comparable perinatal and maternal outcomes. Paper reports on Cochrane review of partograph versus no partograph which found a reduced risk of cesarean delivery in low-income settings (RR 0.38; 95% CI, 0.24–0.61). | Systematic review of observational trials cited in narrative review | ^50^[systematic review], ^51^ [narrative review] | 2+ | May monitor the progress of labor where intrapartum surveillance may be limited by staff shortages and lack of experienced staff | Needs prospective, controlled study, CE assessment |
| Low-cost, low-tech vacuum delivery/EmOC devices | Technology description, no data | Systematic review | ^50^[systematic review], ^51^ [narrative review] | 4 | Can help address logistics and device shortages | No published clinical trials yet |
| Low-cost, low-tech birth simulators | Technology description, no data | Systematic review | ^50^[systematic review], ^51^ [narrative review] | 4 | Needs bundling with training intervention; manufacturers interested in implementation and evaluation | No published clinical trials yet |
| Cell-phone based malaria diagnostics for pregnant women; hemoglobinmeter | Technology description, no data | Narrative review | ^51^ | 4 | Needs to be bundled with malaria services for pregnant women | No published clinical trials yet |
| Clean delivery kits | Births kits are associated with significant increase in attendants having clean hands; intervention packages which include births kits associated with reduced newborn mortality, omphalitis,and puerperal sepsis. Design and use of interventions involving delivery kits, and its implementation are heterogeneous; higher levels of use being reported where birth kits are distributed free as part of a research programme. | Systematic review | ^52, 53^ | 1- | Kits usually part of a larger bundle of interventions, user and training requirement, facilitators and barriers to birth kit use often unclear | Specific effect of kits difficult to isolate; programmes hardly comparable across settings |
| Low-cost EmOC transporter (eRanger) | Reduced median delays in referral to the district hospital by 2·0–4·5 h (35–76%); initial cost of the eRanger was 19-times less than that of a four-wheel drive ambulance based at the district hospital | Narrative review | ^54^ | 4 | Other delays and access barriers to EmOC need to be addressed. | Sustainability of model needs to be assessed. |
| **Neonatal technologies** | | | | | | |
| Low-cost neonatal resuscitators | Innovative low-cost neonatal resuscitation training materials and resuscitation devices in conjunction with a training program suggested a significant reduction in neonatal deaths and rates of fresh still births in Tanzania; and improved provider knowledge, device use, and still birth rates after trainings in India. | Pre-post | ^55^ ^56^ | 3 | Can be bundled with maternal interventions; manufacturers interested in implementation and evaluation | Needs controlled evaluation trials |
| Devices to prevent PMTCT (breastfeeding shields) | Technology description, no data | Narrative review | ^57^ | 4 | Needs integration into PMTCT programmes | Need clinical trials on their acceptability, effect on health outcomes and adverse effects |
| Low-cost, low-tech ventilation devices | Technology description, no data | Narrative review | ^57^ | 4 | Need integration into clinical services; no safety data available | Need clinical trials on their acceptability, effect on health outcomes and adverse effects |
| Low-cost weight or temperature measurement devices | Technology description, no data | Narrative review | ^57^ | 4 | Need integration with clinical services; some clinical data available on temp indicator | Need clinical trials on their acceptability, effect on health outcomes and adverse effects |
| Postnatal clean practice with chlorhexidine cord applications | All-cause neonatal mortality is reduced with chlorhexidine cord applications in the first 24 hours of life, as suggested in trials in Nepal and Pakistan. | Systematic review, cRCT | ^58, 59^ | 1+ | Can be easily integrated into community-based interventions and care at facility | High evidence grade, found to be effective in three cluster RCTs in South Asia |
| Topical application of emmolients to reduce nosocomial infections and associated mortality | Treatment with skin barrier-enhancing emollients (sunflower seed oil or aquaphor) resulted in a improved survival rates among preterm hospitalized infants in Bangladesh. | RCT | ^59^ | 1+ | Part of clean practices that can be integrated into larger MNH programmes | Tested in hospitalized preterm infants, needs studies in term infants |
| Cot-nursing using a heated water-filled mattress for neonatal thermoprotection | Cot-nursing has similar effects to incubator care with regard to temperature control and weight gain in trials from Ethiopia. | Systematic review | ^60^ | 1+ | Electricity dependent | Non-inferiority study; need studies with mortality outcomes |
| Low-cost, low-tech infant warmers | Technology description, no data | Narrative reviews | ^57, 61^ | 4 | Devices marketed and in development; needs bundling with other interventions; manufacturers interested in implementation and evaluation | Need clinical trials on their effect on health outcomes or adverse effects |
| Wraps, foils for neonatal thermoprotection | Technology description, no data | Narrative reviews | ^61^ | 4 | Devices marketed primarily for use in facilities; no distribution channel in low-resource settings | Trials in high-income countries suggest effectiveness, limited data from  low-resource settings |
| Low-cost pulse oximeter | Technology description, no data | Narrative reviews | ^51, 62^ | 4 | Devices marketed and in development | Need clinical trials on their effect on health outcomes or adverse effects |
| Phototherapy devices | Technology description, no data | Narrative reviews | ^63^ | 4 | Devices in development | No published clinical trials yet |

**C. Health Workforce**

| **Innovative MNH approach** | **Summary of Evidence** | | | | **Implications** | |
| --- | --- | --- | --- | --- | --- | --- |
|  | **Evidence** | **Type of Study** | **Reference** | **Grade** | **Programming** | **Implementation Research** |
| **Health Workforce** | | | | | | |
| E-learning | After enrolling in an internet-based distance neonatal care learning offered in India and the Maldives that combined local hands-on skills enhancement, participants had higher skills and knowledge scores and were satisfied with the intervention. | Pre post | ^64^ | 3 | Online trainings are sometimes coupled with on-site skills training, links to other online resources; many more mhealth educational interventions in development (not covered in this review) | Evaluations need to assess not only programme satisfaction or skills and knowledge scores at best- but also cost-effectiveness and impact of e-learning intervention on health of populations served. |
|  | An online training on ENC in India received positive feedback. | Narrative description, feedback | ^65^ | 4 |  |  |
|  | Educational cell phone text messages sent to 2,500 midwives each week for a period of 6 months for continuing education in under-resourced settings in South Africa were well received by midwives in a survey of 50 participants. | Cross-sectional survey on satisfaction | ^66^ | 3 |  |  |
| Training of CHW cadres | Pilot study in Indonesia of community-based neonatal resuscitation training for community midwives and delivery of ventilation devices resulted in improved knowledge and more neonates being ventilated. Newborn survival on day 1 did not differ compared to control group. | Cross-sectional survey with control group | ^67^ | 3 | Implemented in a setting where most deliveries occur at home and almost none of the midwives had previously owned a resuscitation device. | Larger, sufficiently powered studies needed to estimate impact on newborn survival. |
|  | Allowing childbirth companions to support women at state hospital in South Africa during childbirth was not readily accepted by hospitals and did not improve care practices or women's experiences. | RCT | ^68^ | 1- | Childbirth companions alone might not be able to change a health care culture where women were shouted at and report being slapped or struck | Qualitative evaluations might reveal potential modified or alternative strategies to improve care practices and women's experiences |
|  | Evidence on interventions conducted by lay health workers in very varied contexts is limited, but suggests that they provide promising benefits in promoting immunization uptake and breastfeeding, improving TB treatment outcomes, and reducing child morbidity and mortality. NMR is reduced with support of lay workers, but this effect was statistically not significant. | Systematic review | ^69^ | 1- | A variety of models involving CHW have been developed for various regional contexts, some with strong support from countries. Details of CHW models vary among settings, such as training, remuneration or other incentives, and scope of work. | Evidence on effect of CHW on community MNH also available from studies on complex community-based interventions (see Table on “Community participation and mobilization”) |
|  | Community-based neonatal care provided by CHW is associated with reduced NMR, especially in environments with a high NMR before implementation of the intervention. | Systematic review | ^70^ | 1 |  |  |
|  | In Pakistan, training Lady Health Workers links first-level care, including MNH care, to the community. Health indicators are significantly better than the national average in areas served by LHWs | Case study | ^71^ | 3 |  |  |
|  | In India, IMNCI programme training of CHW in community-based MNH care showed intermediate to poor diagnostic agreement, and skills remained poor. | Cross-sectional | ^72^ | 3 |  |  |
|  | With the Morang innovative Neonatal Intervention (MINI) programme in Nepal training CHW to identify and treat severe neonatal infection, treatment was initiated in 90% of cases of suspected severe infection, and CFR was 1.5% in those treated vs 5.3% in those not treated. | Cross-sectional | ^73^ | 3 |  |  |
|  | Training of a cadre of Ethiopian frontline community health workers [Health Extension Workers (HEWs), TBAs and volunteer Community Health Promoters (vCHPs)] in MNH care improved their immediate knowledge scores. | Pre post | ^74^ | 3 |  |  |
|  | A programme of frontline community health workers to improve MH services utilization in Ethiopia increased women's use ANC, but not the rate of facility-based deliveries, skilled birth attendance, or PNC. | Cross-sectional | ^75^ | 3 |  |  |
|  | A novel MNH survival training package for frontline health workers in South Sudan improved knowledge and skills scores, and users were showed satisfaction with the programme. | Pre post | ^76^ | 3 |  |  |
|  | Teaching sessions in the Philippines training male community representatives in MNH interventions improved their knowledge scores. | Pre post | ^77^ | 3 |  |  |
|  | Case management and support by dedicated CHWs can create a continuum of longitudinal care in the PMTCT cascade, improve testing and treatment initiation, and support retention in PMTCT care. | Cross sectional | ^78^ | 3 | Other community-based case-management models involve TBAs or other cadres. | Evaluation of impact of case management approach on health impact needed. |
|  | Home management of (maternal) malaria in Zambia costs less than facility-based treatment and is more cost effective. | Costing study | ^79^ | 3 |  |  |
|  | Two categories of approaches are likely to increase the use of skilled health personnel at birth: a) deploying skilled health personnel and b) addressing financial barriers for users. | Systematic review | ^80^ | 1+ | Integral approach beyond workforce interventions needed to improve skilled birth attendance. | Improved access to skilled health personnel for childbirth shown to have positive outcomes for MNH indicators |
| **Task shifting to non-physicians** | | | | | | |
| Non-physician clinicians to provide EmOC | Non-physician clinicians did not differ in key clinical outcomes of OB surgery when compared to physicians. Wound infections and dehiscence were more common when surgery was done by non-physicians. | Systematic review | ^81^ | 1+ | Various models implemented in Africa (Zaire, Mozambique, Malawi, Burkina Faso, Tanzania) | Studies are not randomized nature, and do not assess risk of cases assigned to officers vs physicians. |
|  | In Tanzania, competency-based curricula for assistant medical officers’ (AMOs) training in CEmOC, and for nurses, midwives and clinical officers in anaesthesia and operation theatre etiquette (involving hands-on sessions, lectures and discussions; monthly supportive supervision) led increase in surgeries at institutions where teams of non-physician providers had been trained, while SBR and referrals for OB care decreased. | Pre post | ^82^ | 3 | Although officers performed most of cesarean deliveries in study areas in Tanzania, met need was only between 23 and 35%. |  |
|  | In Tanzania, most surgeries were performed by non-physicians, with a CFR of 1.2 to 2%, close to the 1% target for safe EmOC. | Cross sectional | ^83^ | 3 |  |  |
| Task shifting of mid-level anaesthesia services | Task shifting expands coverage and access to OB care in South Asia, but has not been evaluated or scaled up yet. | Narrative review | ^84^ | 4 | Anaesthesia task shifting might expand coverage and access to care in South Asia | Systematic safety and health outcomes evaluation needed. |
| Newborn aides to staff NICU | In India, in the context of lack of nurses, a cadre of newborn aides might help staff newborn care units. | Narrative review | ^85^ | 4 | Novel cadres might help bridge personnel gap ininstitutions |  |
| Pictorial job aids used by skilled providers and for task shifting to clinic-based lay providers | Pictorial job aids increase certain kills and knowledge scores and can improve counseling on routine ANC and ENC in a pilot study in Benin. | Pre post with control group | ^86^ | 3 | Pictorial aids might help shift tasks to lay nurse aides to provide effective antenatal counseling in facility-based settings | Need to assess how to tailor aids to the counseling situation and target audience (use of aids did not change maternal knowledge of general prenatal or newborn care). |
|  |  |  | ^87^ | 3 |  |  |
|  | With pictorial aids, more messages were provided during counseling, and more women had knowledge of key messages of perinatal health in a pilot study in Benin. | Report | ^88^ | 4 |  |  |
| **Training health providers** | | | | | | |
| Low-tech OB simulation training programme | A simulation-based EmOC and neonatal care training (PRONTO) in Mexico helped reach hospitals goals and increased knowledge scores. | Cross sectional | ^89^ | 3 | Based on low-tech simulation, improved knowledge scores. | In addition to attainment of hospital goals such as reaching logistic goals, need to measure skills and health impact. |
| Simplified training to Improve Neonatal Resuscitation—Helping Babies Breathe Programme | The HBB programme adopts US resuscitation guideline for use in resource-limited settings. | Narrative review | ^90^ | 4 | Training standards from industrialized context was adapted to training needs in low-resource settings | Additional evidence available from more recent studies (see Table “Survey’) |
|  |  | Report | ^91^ | 4 |  |  |
| Training of health professionals and policy-makers in essential neonatal care and breastfeeding promotion | WHO programmes such as Making Pregnancy Safer and Promoting Effective Perinatal Care have led to positive changes even in challenging contexts. | Narrative review | ^92^ | 4 | Simple cost-effective interventions aimed at improving quality of healthcare in former Soviet countries | No formal programme evaluation published. |
| Train-the-trainer model for instruction in the WHO ENC course and neonatal resuscitation | In a pre post evaluation, training in the WHO ENC course did not change NMR, but decreased SBR. In an RCT, resuscitation training did not show an effect on NMR or SBR. | RCT, pre post | ^93^ | 1+ | Implemented in various countries globally. | Might need an evaluation of morbidity with ENC and neonatal resuscitation |
| Training of health care providers (WHO ENC course) | Early NMR decreased after ENC training of clinic midwives in Zambia. | Pre post | ^94^ | 3 | The intervention costs were $208 per life saved and $5.24 per disability-adjusted life-year averted. | Need to assess potential for scale-up. |
|  | Training of midwives in Zambia using the WHO ENC course improved knowledge and skills scores. | Pre post | ^95^ | 3 | Application of the ENC guidelines contingent on basic resources; implementation of the ENC course needs to consider local conditions available | Study methods tailored to training materials, but no data on long-term skills or knowledge retention, or health impact. |
|  | ENC training of midwives and implementation among mothers at 18 centers was associated with a decrease in early NMR, particularly among mothers with limited school education. | Pre post | ^96^ | 3 | The impact of ENC may be optimized by training health care workers who target women with less formal education. | Needs data on long-term skills or knowledge retention, or health impact. |
| Training Providers to Improve Neonatal Resuscitation | The China NRP trained 110,000 professionals and reached 94% of birthing facilities in 99% of the counties studied. "Intra-partum related deaths" decreased during the time period of implementation. | Interrupted time series | ^97^ | 3 | Implemented in 20 provinces. | Given the policy changes achieved by Chinese NRP, need to assess population-level mortality and morbidity |
| Training of health care providers for mother and newborn health care improvement through international federation | The FIGO saving mothers and newborns projects aim to partner professional associations across countries. | Narrative review | ^98^ | 4 | Projects are developed by partnering professional associations between high- and low-resource countries. | Pilots in various countries assessed knowledge and skills scores (data not given in this ref). |
| Training in acute care of at-risk newborns | The adaptation of a Canadian training programme for Chinese practitioners was well received and increased knowledge and confidence in newborn care in China. | Pre post | ^99^ | 3 | International program adaptation requires structured and systematic evaluation to ensure that the programme meets the needs of learners, reflects their learning styles, and can be applied in their setting. | |
| IMNCI programme training CHW in community-based MNH care | Modifying the IMNCI training into smaller training units reduced training costs, while improving knowledge and skills compared to baseline. | Pre post with controls | ^100^ | 3 | Modifications saved US$813 for a training of 25 and indirect cost saving of 3 days per trainee. | Might need to assess long-term practices, health impact, and potential for dissemination of model |
|  | When implemented in India, the programme was hampered by inadequate supervision and supplies. There was no statistical difference between districts except higher care seeking for ARI in intervention districts. | Pre post with control districts | ^101^ | 3 | Rapid programme assessment was conducted to aid training of 200,000 community health workers across 223 districts. | Evaluations need to assess aspects that can inform improved implementation strategies (supportive supervision, supply logistics, and monitoring) |
| Educating nurses using QI methods to improve neonatal health | A quality improvement project to provide nurses with the training and tools to decrease neonatal mortality and morbidity. | Study Protocol | ^102^ | 4 | n/a | n/a |
| Training health care providers in newborn care | In a pilot study in Sri Lanka, a 4 day training programme led to improvements in practices such as thermal protection, early initiation of breastfeeding, and hygiene. | Pre post with control | ^103^ | 3 | Short training had an impact on observed newborn care behavior. | Might need to assess long-term practices, health impact, and potential for dissemination of model |
| Perinatal Continuing Education Programme | The adaptation of a perinatal training programme led to increased knowledge in perinatal care in regional hospitals in Mexico. | Pre post | ^104^ | 3 | Programme based on self-teaching and participation in skills demonstration and practice sessions. | Need to assess health impact, long term knowledge retention. |
| Training of health care providers in EmOC | Most studies report positive reactions, increased knowledge and skills, and improved behaviour after training. Outcome is assessed less frequently, and positive effects are not always demonstrated. | Narrative review | ^105^ | 4 | Effective training approaches often include practical components, team work, and follow-up trainings. | According to various levels of evaluations (Kirkpatrick framework), training programmes vary in design and are often inadequately evaluated. |
|  | A national training programme increased the number of facilities providing EmOC in Bangladesh. | Narrative review | ^106^ | 4 | Competency-based training, innovative incentives to retain trained staff, and standardised evidence-based protocols part of training | Need to assess health impact, long term knowledge retention. |
|  | Following the training in India, basic EmOC skills were performed more often than before, and facilities to which trainees returned were able to offer EmOC services. | Narrative review | ^107^ | 4 | Training needs to address insufficient training for cesarean delivery, lack of anesthetists, equipment and infrastructure |  |
| Training of maternity auxiliary nurses | Educational conferences for auxiliary nurse midwives, taught by external instructors in the Dominican Republic have developed into activities of an NGO aiming at building capacity. | Narrative review | ^108^ | 4 | Implementation and sustainability build on local ownership. | No formal programme evaluation published. |
| Training of SBA and centralization of EmOC and neonatal services | While German NGOs provided support and maternal care was centralized in Eritrea, national MMR rates declined | Narrative Review | ^109^ | 4 | Approach included both trainings and centralization of care. | No formal programme evaluation published. |
| Essential Surgical Skills-Emergency Maternal and Child Health (ESS-EMCH) Programme | The Essential Surgical Skills - Emergency Maternal and Child Health in Pakistan has led to improvements in MNH care | Narrative Review | ^110^ | 4 | Certification was felt important to assure quality | No formal programme evaluation published. |
| Training of health care providers in ANC to provide community-based care | Knowledge scores increased after the training, and enrolment of pregnant women in ANC increased from a mean of 2.2 times per pregnancy to 3.4 times. | Pre post | ^111^ | 3 | Training methods included role-playing, demonstrations using visual information, and hands-on practice. | Need to assess health impact, long term knowledge retention. |
| **Training TBAs** | | | | | | |
| Training TBAs in ANC, safe delivery, and newborn care | In several cRCTs, training of TBAs was shown to be associated with reductions in PMR and NMR; reductions in MMR were statistically non-significant | Systematic review | ^112^ | 1++ | Strategies incorporating training and support of TBAs can reduce perinatal and neonatal deaths | Available data is from randomized and non-randomized studies |
|  | cRCT have been shown TBA trainings to reduce PMR and NMR in clusters of trained TBAs when compared to untrained TBAs. | Systematic review | ^113^ | 1+ |  |  |
| Training of TBAs in neonatal resuscitation, early recognition and treatment of infections | In rural Zambia, mortality at day 28 after birth was 45% lower among live born infants delivered by intervention birth attendants. | cluster RCT | ^114^ | 1+ | Similar programme components might be effective in other rural settings. | Sustainability of training to be determined. |
| Training of TBAs in ENC (WHO programme) | In the period following the training in Guatemala, PMR and SBR decreased | Pre post | ^115^ | 3 | Pilot conducted in 11 rural areas. |  |
| Training of TBAs in ENC (WHO programme), followed by resuscitation training | In the Congo, training TBAs in ENC did not change PMR; early NMR declined in the year after the ENC training, but neonatal resuscitation training showed no effect on mortality. | Pre post | ^116^ | 3 | A period of re-enforcement of training may be necessary to show impact on mortality. | Needs further evaluations to assess potential for scale-up. |
| Training TBAs in ANC, safe delivery, and newborn care ("SMART Dai" Method) | In Pakistan, knowledge and skills scores were higher among TBAs in the trained cluster, with an effect shown after 1.5 years. | RCT | ^117^ | 1- | Intervention implemented in 120 rural communities each with a population of approximately 5000. | Study compared areas with community-based intervention versus health systems intervention |
| Training TBAs in ANC, safe delivery, and newborn care | Self-reported practices improved after training sessions in Bangladesh. Greater effects (improvement, higher scores) were observed among TBAs with no prior training. | Pre post | ^118^ | 3 | Trainees' prior experiences and beliefs may affect knowledge acquisition. | Need to assess health impact, long term knowledge retention. |
| Training of TBAs in newborn care | In India, knowledge and skills scores improved after the training. There were fewer deaths observed after the training. | Pre post | ^119^ | 3 | Training programmes for TBAs need regular reinforcements. | Needs adequately powered evaluation studies. |
| Training of former TBAs as clinic-affiliated maternal health workers | In Lesotho, after an NGO training for TBAs, visits recorded by the clinic increased from 20 to 31 monthly, and institutional deliveries increased from 46 to 216 yearly. | Narrative | ^120^ | 4 | Implemented by well-established international NGO (Partners in Health) | No formal programme evaluation published. |
| Training matrones (auxiliary midwives) in oxytocin use | NGOs such as PATH seem well positioned to implement approaches such as training auxiliary health cadres in oxytocin use. | Narrative review | ^121^ | 4 | NGOs can use and expand research to help shape appropriate interventions for diverse communities. | N/a |
| Training TBAs in the use of absorbent delivery mat and misoprostol use | TBAs in Bangladesh used the mat and misoprostol correctly. Knowledge scores improved, an effect which remained 18 months after the training. | Pre post | ^122^ | 3 | PPH management might be feasible in home births using misoprostol and the blood collection delivery mat | Need to assess health outcomes. |

**D. Health Financing**

| **Innovative MNH approach** | **Summary of Evidence** | | | | **Implications** | |
| --- | --- | --- | --- | --- | --- | --- |
|  | **Evidence** | **Type of Study** | **Reference** | **Grade** | **Programming** | **Implementation Research** |
| **Financial Incentives** | | | | | | |
| Conditional Cash transfers | Aama (Mothers’) Programme (cash transfer element) in Nepal; the Janani Suraksha Yojana (Safe Motherhood Scheme) in India; the Chiranjeevi Yojana (Scheme for Long Life) in India; the Maternal Health Voucher Scheme in Bangladesh and the Sehat (Health) Voucher Scheme in Pakistan increased use of maternal health services. | Case study | ^123^ | 4 | Areas for improvement in these schemes are the need for more efficient operational management, clear guidelines, financial transparency, plans for sustainability, evidence of equity. | Evidence of improvements in health outcomes has not been established due to a lack of controlled studies. Needs data on impact on quality of care, mortality and morbidity. |
|  | Janani Suraksha Yojana program, conditional upon either in-facility delivery or skilled birth attendance | Interrupted time series and qualitative | ^124^ | 3 |  |  |
|  | MATIND study protocol evaluating Janani Suraksha Yojana and Chiranjeevi Yojana ( voucher based program to reimburse private obstetricians in Gujarat) | Protocol | ^125^ | 4 |  |  |
| Nepal Safe Delivery Incentive Programme | 1) Cash incentives to women for attending a facility for delivery, varying by geography, to help offset charges at facility, 2) Free delivery in the 25 poorest districts, and 3) Incentive to skilled birth attendants for delivery. | Case study | ^126^ | 4 | Prompt policy acceptance and implementation following research and communication of results responding to both technical and political policy-making concerns. | Needs data on impact on quality of care, mortality and morbidity. |
|  | SDIP had modest effect on the utilization of maternity services. Women who had heard of the SDIP before childbirth were 4.2 percentage points (17 %) more likely to deliver with a skilled attendant. | Cross sectional | ^127^ |  | Coverage was low, and effects associated with the size of the financial incentive and the quality of care in facilities. |  |
| Performance-based payment in Rwanda | Pay-for-performance was associated with a significant increase (23%) in the probability of a woman delivering in a facility (and of a child visiting a facility for preventive care), but no effect on the number of prenatal care visits. | RCT | ^128^ | 1++ | Incentive design (size, frequency ect.) needs to be tailored to target population. | Needs data on impact on quality of care, mortality and morbidity. |
| Performance-based payment in the D.R. of the Congo | In the performance- based financing districts, more childbirth occurred in a facility. | Pre post with control | ^129^ | 3 | Performance-based financing mechanisms seem feasible even in frail states. | Needs data on impact on quality of care, mortality and morbidity. |
| **Voucher for services** | | | | | | |
| Voucher for maternal health services | Adequate administrative and financial resources for timely processing and disbursement of vouchers and incentive payments, and contextually appropriate and understood selection criteria for enrolment are needed for successful programmes in Bangladesh. | Qualitative | ^130^ | A | Where local health service capacity is limited, a demand-side strategy might need significant expansion of the service delivery capacity of health facilities. | Needs data on impact on quality of care, mortality and morbidity. |
|  | Defined benefit packages, contracting and quality assurance; marketing and distribution of vouchers and claims processing and reimbursement were key steps in Kenya. Programmes need effective marketing with adequate information for clients on the benefit package. Government’s role should include provision of adequate funding, stewardship and scale up of such voucher programmes. | Qualitative | ^131^ | B | Implementation challenges included limited feedback to providers on the outcomes of quality assurance, accreditation and budgetary constraints. | Needs data on impact on quality of care, mortality and morbidity. |
|  | Institutional delivery rate increased among women in the fourth or fifth wealth quintiles in the intervention union councils in Pakistan, while no significant changes in the control union councils. Increase in institutional delivery among poor women relative to non-poor women was significantly greater in the intervention compared to the control union councils. | Pre post with control area | ^132^ | 3 | Demand-side financing projects using vouchers can be an effective way of reducing inequities in institutional delivery. | Needs data on impact on quality of care, mortality and morbidity. |
|  | Women who were sold voucher booklets were significantly more likely than women who delivered the year before implementation to make at least three ANC visits, deliver in a health facility, and make a postnatal visit (purchase of a voucher booklet associated with a 22 % point increase in ANC use, a 22 % point increase in institutional delivery, and a 35 % point increase in PNC use). | Cross sectional (with sampling strata of mothers reporting deliveries the year before and during programme implementation) | ^133^ | 3 |  |  |
|  | Trial investigated effects of residing in voucher sub-districts on the use of professional maternal health services and associated out-of-pocket expenditures in 16 intervention and 16 matched comparison sub-districts. Significantly increased use of antenatal, delivery, and postnatal care with qualified providers in sub-districts with voucher programmes in Bangladesh. Women in programme areas paid less for maternal health services. No significant effect of vouchers on the rate of Cesarean section. | Non-random controlled trial | ^134^ | 1- |  |  |
|  | After voucher programme implementation in Cambodia, significant increase in facility-based deliveries, while proportion of women completing 4+ ANC visits significantly lower. Multiparous women, those with four or more children and those classified as the ‘least poor' less likely to purchase voucher. | Interrupted time series | ^135^ | 3 |  |  |
|  | Protocol on the impact of the voucher and accreditation approach on improving reproductive behaviors and status in Cambodia | Protocol | ^136^ | 4 |  |  |
| Vouchers for maternal health services and for transportation costs; and gift box and a cash payment to women who deliver at a health facility | Voucher recipients (for 3 ANC check-ups, safe delivery at a facility or at home by skilled birth attendants, PNC, EmOC) in the project area were 2.0 times more likely to get ANC, 3.6 times more likely to have skilled birth attendance, 2.5 times more likely to deliver in a facility, 1.5 times more likely to seek treatment for obstetric complications, 2.8 times more likely to receive PNC than those not in the program. | Non-random controlled trial | ^137^ | 1- | The use of vouchers has stronger demand-increasing effects on the poor, but SE disparity remains. | Needs data on impact on quality of care, mortality and morbidity. |
| Vouchers for maternal health care services and treatment of pregnancy and delivery related complications, transport costs. | Has additional cash incentive for delivery at facility or at home in presence of skilled birth attendant. | Protocol | ^138^ | 4 | n/a |  |
| Vouchers for maternal health services and transportation costs for pregnant women. | "Increase" in facility based deliveries in areas in Bangladesh with voucher programme; no increase in surgical deliveries. | Interrupted time series with controls; and qualitative | ^139^ | 3 | Despite additional funding to facilities, remains complex to administer, requiring a parallel administrative mechanism putting additional work burden on the health workers. | Needs data on impact on quality of care, mortality and morbidity. |
| Vouchers for maternal health services and transportation costs for pregnant women; financial incentive from government in addition to user fee for birth attendants | Facility deliveries increased over 2 years from 16% pre to 45% post introduction of voucher and health equity funds (HEF) schemes, including voucher and HEF beneficiaries, but also selfpaid deliveries (increase by 29% compared to 15% and 9% increases in non-voucher areas; no statistcal significance given). | Cross sectional and qualitative | ^140^ | 3 | Need other interventions to ensure the supply of sufficient quality maternity services and to address other non-financial barriers to demand. | Needs data on impact on quality of care, mortality and morbidity. |
| Vouchers for motorcycle transport and for payment to service providers for antenatal, delivery, and postnatal care; and health worker refresher training, additions of minimal drugs and supplies | Limited report of results: rates of deliveries in health facilities in Uganda and first postnatal care visits per month increased in intervention vs control areas | Non-random controlled quasi-experimental trial | ^141^ | 1- | Trial still ongoing. | Needs data on impact on quality of care, mortality and morbidity. |
| Vouchers for RH, including maternity services | In Bangladesh and Cambodia, facility-based deliveries had a greater increase in voucher areas compared to control areas (Bangladesh also significant increases in antenatal and post-natal care visits compared to controls). | Systematic Review | ^142^ | 2+ | All evaluations reported some positive findings, indicating that RH voucher programmes increased utilization of RH services, improved quality of care, and improved population health outcomes. | No studies to examine programme effectiveness using strong study designs, and there is no evidence on cost-effectiveness and population health impacts. |
| **Community-based health insurance schemes** | | | | | | |
| Community-based health insurance | Membership in an insurance scheme was positively associated with use of maternal health services, particularly in areas in West Africa where utilization rates are very low and for more expensive delivery-related care. | Cross sectional | ^143^ | 3 | Inclusion of maternal health care in insurance benefits package is key. Complementary supply-side interventions to improve quality of and geographic access to health care are also critical. | Needs data on impact on quality of care, mortality and morbidity. |
|  | While insurance coverage in Rwanda’s Mutuelles (community-based health insurance programmes) increased from 1% to 85% during implementation period, skilled birth attendance increased from 39 to 67%. | Interrupted time series | ^144^ | 3 | Can be implemented even in the poorest settings. | Needs data on impact on quality of care, mortality and morbidity. |
|  | With the implementation of Mutuelles in Rwanda, use of assisted deliveries increased from 12% to 43% among the poorest quintile. (Utilization of modern health care providers among children under 5, suffering from fever/cough increased from 13-22%.) | Interrupted time series | ^145^ | 3 |  |  |
|  | During implementation of the insurance scheme (NCMS) in China, having no any pre-natal visit decreased from 25% to 12%; facility-based delivery increased from 45% to 80% | Interrupted time series | ^146^ | 3 | Although participation reduced out-of-pocket payments, the rural poor were still facing substantial payment for facility-based delivery. | Needs data on impact on quality of care, mortality and morbidity. |
| Obstetric insurance | Most (95%) of pregnant women in the catchment area in Nouakchott, Mauritania (covering 48.3% of the city's deliveries) enrolled. Facility utilization rates increased over the 3-year period of implementation. | Pre post | ^147^ | 3 | The program has generated more than twice as much in revenues as current user fees. | Needs data on impact on quality of care, mortality and morbidity. |
| **User fees** | | | | | | |
| User fee abolition in Ghana | Fee exemption mechanisms well accepted and context-appropriate. Staff workloads increased as more women attended, and levels of compensation for services and staff were important to the scheme’s acceptance. | Qualitative | ^148^ | C | Initial problems with disbursing and sustaining the funding, and with budgeting and management. | Needs data on impact on quality of care, mortality and morbidity. |
|  | Compared to before fee exemption, proportion of women delivering in health facilities increased in each income quintile after fee abolition. | Interrupted time series | ^149^ | 3 |  |  |
| Various interventions such as user fee abolition, insurance, vouchers , transport cost loan funds, CCT | For removal of user fees and provision of universal coverage for pregnant women to be successful, governments must replenish the income lost. Maternal health care needs are to be included in insurance benefits packages. | Narrative review | ^150^ | 4 | Available evidence creates a strong case for removal of user fees and provision of universal coverage for pregnant women. | Effectiveness of voucher schemes, methods to target financial assistance for transport and time costs to be tested in LMIC. |
| User fee reduction or abolition | In combination with community health insurance, vouchers and health equity funds for obstetric care, and incentive payments for delivery in health facilities, user fee interventions need local commitment, perseverance and adaptability, a holistic approach addressing demand- and supply side barriers, and a focus on universal coverage key to succeed. | Narrative review | ^151^ | 4 | Shown to increase service utilization increased in most of the settings. | Need to address quality of care, equity between rich and poor patients, and financial sustainability. |
| User fee cost-sharing in a district hospital in Burkina Faso | Cost-sharing system covering pregnancy emergencies, transportation fee, for a payment of 25000 FCFA (46US$), rest shared by health centres, Ministry of Health and local authority led to increase in rates of major OB interventions. | Interrupted time series | ^152^ | 3 | With this scheme, at cost recovery rate of 91%, the balance at programme end was positive. | Need to address quality of care, equity between rich and poor patients, and financial sustainability. |
| User fee reduction offering an 80% subsidy for facility-based delivery in rural Burkina Faso | Over 5 years of program activities, the proportion of facility-based deliveries increased from 49 to 84 %. The utilization gap across socioeconomic quintiles, however, remained unchanged. | Interrupted time series | ^153^ | 3 | In spite of subsidy, women continued to pay on average more than the set tariff. | Needs data on impact on quality of care, mortality and morbidity. |
| Fee reimbursement: mix of 1) Reimbursement of delivery costs for mothers, 2) clinical training sessions for hospital providers, 3) training of township midwives and village doctors/ family planning workers , focused on communication and health promotion skills | Between-group differences were small and varied between study locations (provinces in rural China); financial intervention not associated with number of visits, but with increased caesarean sections and decrease in ultrasound tests. Clinical training led to increase in some indicators of care content. | cRCT | ^154^ | 1- | Implementation challenging. Concerns with programme included unintended consequences, such as overuse of technology. | Needs data on impact on quality of care, mortality and morbidity. |

**E. Community ownership and participation**

| **InnovativeMNH approach** | **Summary of Evidence** | | | | **Implications** | |
| --- | --- | --- | --- | --- | --- | --- |
|  | **Evidence** | **Type of Study** | **Reference** | **Grade** | **Programming** | **Implementation Research** |
| **Mothers and Women Groups** | | | | | | |
| Monthly mothers group activities to improve perinatal health in Makwanpur, Nepal, convened by female facilitators who supported groups through an action-learning cycle in which they identified local perinatal problems and formulated strategies to address them. | MMR was much lower in intervention clusters with the women’s groups intervention than in control clusters, and women were more likely to have antenatal care, institutional delivery, trained birth attendance, and clean care. | cRCT | ^155^ | 1++ | The intervention cluster had an average population of 7000. Of the 111 women's groups formed for the Makwanpur trial, 100 groups continued to meet after the study was completed after 2.5 years. | Similar community participatory activities to be tested in other settings. |
|  | These groups developed innovative approaches to MNH, such as MNH funds and production of clean delivery kits. | Narrative Review | ^156^ | 4 |  |  |
| Women group activities to improve perinatal health | Facilitators in Mumbai, India, supported women's groups through an action learning cycle in which they discussed perinatal experiences, improved their knowledge, and took local action. There were no differences in uptake of antenatal care, institutional delivery, breastfeeding, care-seeking, or perinatal mortality rate. The stillbirth rate was non-significantly lower and the neonatal mortality rate higher. | cRCT | ^157^ | 1+ | Community mobilization might facilitate behavior change, but additional; intervention might be necessary to reduce mortality. | Although the study covered a population of 283,000, it did not detect mortality effects. |
| Scaling up of women group activities to improve perinatal health | In the study districts in Bangladesh, facilitators convened 18 groups every month to support participatory action and learning for women, and to develop and implement strategies to address MNH problems. Participatory women's groups had no statistically significant effect on NMR. | cRCT | ^158^ | 1+ | For community participation to have an effect on mortality, enhanced coverage and increased enrolment of newly pregnant women might be needed. | All study areas, including controls, received health services strengthening and basic training of traditional birth attendants. |
| Female CHW outreach and women group activities to improve perinatal health | In Sylhet, Bangladesh, home-care by female CHW who identified pregnant women, made two antenatal home visits to promote birth and newborn-care preparedness, made postnatal home visits to assess newborns, and referred or treated sick neonates was compared to community-care, where birth and newborn-care preparedness and care-seeking were promoted solely through group sessions held by community mobilisers. Compared to control areas, home care reduced NMR by about a third. Community-care and women group activities had no statistically significant effect on NMR. | cRCT | ^159^ | 1+ | Implementation might  need to be tailored to areas with high poverty, poor availability  and access to health services, and a general resistance in  taking newborns and postpartum mothers outside of the  home for treatment. | Implementation  of the community-care strategy for an increased  period might be needed to improve coverage  and show an effect on mortality? |
| Mothers group activities to improve perinatal health in Nepal | In Nepal, there was minimal level of involvement of community networks. Female community health volunteer were engaged in delivering messages at household level rather than mother's groups, who had been least engaged in identifying and solving MNH problems. | Qualitative study | ^160^ | C | The Community Action Cycle methodology could be used to engage mothers groups in MNH activities. | Experiences in Nepal suggest that alternative methods need to be explored to implement community participation and mobilization strategies. |
| **Complex Interventions** | | | | | | |
| Community-based intervention packages to reduce maternal and neonatal morbidity and mortality | Meta-analysis of data from RCTs suggests that community-based intervention packages do not reduce maternal mortality, but improve maternal morbidity, still births, and neonatal mortality. They also improve referral to health facilities, and breastfeeding rates. | Systematic literature review | ^161^ | 1+ | Data supports the value of community-based care for MNH through a range of interventions which can be packaged effectively for delivery facilitated by community participation and mobilization. | Need to assess additional components addressing maternal mortality. |
| Home-based neonatal care (HBNC) and health education in Gadchiroli | Home-based care and health education reduced the incidence of neonatal morbidities and low birth weight in Gadchiroli, India. Maternal knowledge and behaviors also improved during that time. | Pre post with control areas | ^162^ | 3 | Interventions as part of the package were increasingly implemented over time. | Intervention was specifically designed for and with community in intervention area |
|  | The Gadchiroli trial suggests that a low-cost approach is feasible to address newborn health at the community level. | Commentary | ^163^ | 4 |  |  |
| Shivgarh community-based intervention package | The community-based intervention in Shivgarh, India, focused on the provision of ENC and on prevention of newborn hypothermia, and was associated with a reduction in NMR by about 50%. The intervention included birth preparedness, clean delivery and cord care, thermal care (including skin-to-skin care), breastfeeding promotion, and danger sign recognition; some received a liquid crystal hypothermia indicator (ThermoSpot). | cRCT | ^164^ | 1+ | Community health workers delivered the packages via collective meetings and two antenatal and two postnatal household visitations. | The interventions were socioculturally contextualized in the given setting, targeted at high-risk newborn-care practices |
| Indian Integrated Management of Neonatal and Childhood Illness (IMNCI) | In the IMNCI programme in Haryana, CHW were trained to conduct postnatal home visits and women's group meetings; in addition, physicians, nurses, and CHW were trained to treat or refer sick newborns and children. This reduced NMR beyond 1st day and IMR. Newborn care practices also improved with IMNCI. | cRCT | ^165^ | 1+ | The data provides evidence to scale-up IMNCI as part of India's MNH strategy | Further evaluations to assess IMNCI effect in other settings. |
| Community-based newborn care package integrated into national health system in Nepal | Nepal integrated a community-based newborn care package (CB-NCP) into the existing structures of their health system. | Narrative Review | ^166^ | 4 | Extend of community participation and mobilization unclear. | See Table “Policy approaches” |
| Networks model: group-based outreach activities in Uganda | The project's outreach work to more than 1 million people organized and mobilized individuals living with HIV and their families to strengthen HIV prevention and care within their communities. | Narrative Review | ^167^ | 4 | The model facilitated coalition building to improve referral and literacy activities; and reach and coverage of HIV services through strengthened linkages with healthcare facilities. | Process and impact evaluations not yet published. |
| Mirzapur community-based intervention of antnatal and postnatal visits from CHW | In the intervention in Mirzapur, Bangladesh, CHW identified pregnant women; made two antenatal and four postnatal home visits; referred sick neonates to a hospital and facilitated compliance. Indicators of care improved, but mortality did not change with the intervention. Given the high program coverage and quality assurance of implementation, this might imply risk factors for mortality need to be addressed with more specific interventions. | cRCT | ^168^ | 1+ | The cause-structure of neonatal mortality needs to be factored in when developing interventions. This intervention focused on essential newborn care and infection prevention and management. | Future evaluations might assess management of birth asphyxia and prematurity, and curative early postnatal care. |
| MOM project: network of community-based providers for MH | In Burma, health workers from local organizations received practical training in basic emergency obstetric and antenatal care; they trained a second tier of local health workers and a third tier of traditional birth attendants (TBAs) (also see Table “Workforce – Task Shifting). | Narrative Review | ^169^ | 4 | Close communication between health workers and TBAs promoted acceptance and coverage of maternity services throughout remote communities. | Capacity building with community participation might serve as a model to be evaluated in other conflict settings. |
|  | After the implementation period, pregnant women were more likely to receive ANC, skilled birth attendance, and PNC. | Pre post | ^170^ | 3 |  |  |
| Hala community-based intervention package delivered by lady health workers, who focused on essential maternal and newborn care, conducted community education group sessions, and were encouraged to link up with local Dais | After implementation of the pilot intervention, SBR and NMR were significantly reduced. | Pre post | ^171^ | 3 | The intervention was delivered within the regular government LHW programme and was supported by the creation of voluntary community health committees. | Evaluation suggest that preventive and promotional maternal and newborn interventions through CHW can be scaled-up |
|  | In intervention clusters, SBR and NMR were lower than in control clusters. | cRCT | ^172^ | 1+ |  |  |
| Community-based MNH care package delivered through trained local female facilitators | This community mobilization intervention, implemented in Mchinki district in Malawi, involved women's groups to build the capacities of communities to take control of the mother and child health issues that affect them. | Narrative review | ^173^ | 4 | The intervention uses a manual, participatory appraisal tools, and visual aids to catalyze community action for MNH. | Useful description, health impact evaluation not published. |
| CHW delivered community-based newborn care package (Manoshi programme) | In a project in Dhaka, Bangladesh, the lack of financial incentives was a barrier, social prestige and positive feedback were facilitators for CHW remaining active. | Cross sectional survey and qualitative (IDIs) | ^174^ | 3 | Study focused on CHW retention; extend of community participation and mobilization unclear. | Health impact evaluation not published. |
| CHW-delivered community-based OB care package | After training of 50 safe motherhood promoters (SMP) in Mtwara, Tanzania, skilled attendance increased from 34 to 51%, and early ANC bookings from 19 to 57%. SMPs closely collaborated with existing community structures and health services. | Pre post | ^175^ | 3 | Implemented in 4 villages with a total population of 8300. | Health impact evaluation not published. |
| Community-based quality improvement processes | Protocol to test whether in Vietnam, a facilitation intervention on the community level, with a problem-solving approach involving local representatives if the healthcare system and the community, results in improvements of neonatal health and survival. | Study Protocol | ^176^ | 4 | The approach focuses on developing a learning process and a problem-solving cycle with the local community as actors in newborn health, based on existing healthcare structures. | Also see Table “Health services – quality improvements” |
| Community-based intervention packages (Manoshi) to reduce maternal and neonatal morbidity and mortality through early referrals to EmOC | In in urban slums of Bangladesh, a woman-focused development intervention emphasized timely referral of the obstetric complications and reduced delays in accessing EmOC. Women cared for at delivery centers presented with reduced time for making the decision to seek care for complications compared to mothers who were referred from home. Reasons for first delay included fear of medical intervention, inability to judge maternal condition, traditional beliefs and financial constraints. Financial assistance reduced delays. | Cross sectional survey | ^177^ | 3 | Extend of community participation and mobilization unclear. | Health impact evaluation not published. |
| Scaling up a community-based intervention: cycles of women's groups meetings on MNH led by a facilitator | The intervention could be scaled up from 162 to 810 woman's groups in rural Bangladesh, without financial incentives for communities or increase in managerial staff. Scale-up requires programmatic and operational flexibility. | Narrative review | ^178^ | 4 | Strong operational capabilities and institutional knowledge of the implementing organisation were critical to the success of scale-up. | Monitoring and feedback systems for periodic programme corrections and continued innovation were central to successful scale-up. |
| Community health promotion in Andra Pradesh (India) through a package of MNH care interventions (women's groups, CHW training) | The intervention includes a health promotion campaign, participatory discussion groups, training of village health workers and midwives, and improved coordination of antenatal services. The intervention group will also have subsidized access to pregnancy-related healthcare services at non-public health centres . | Study Protocol | ^179^ | 4 | The intervention combined a community health promotion campaign and a system to contract out healthcare to non-public institutions. | n/a |
| Home-based newborn health care in Brazil | Women in this study viewed home-based newborn care as positive. | Qualitative Study | ^180^ | C | Extend of community participation and mobilization unclear. | Health impact evaluation not published. |
| **Home-based care facilitated by communities** | | | | | | |
| Home-based life-saving skill building programme (HBLSS) of community-based guides training pregnant women, caregivers, and birth attendants | Programme-trained guides performed better in the management of PPH. About 38% of pregnant women were exposed to the programme. | Pre post; IDI to review cases with complications | ^181^ | 3 | HBLSS was field tested in rural southern Ethiopia where over 90% of births take place at home with unskilled attendants. | Women giving birth were exposed to HBLSS training; but community participation unclear. Health impact evaluation not published. |
|  | Follow-up evaluation of above study. Programme-trained guides performed better immediately and 1 year after training. About 54% of pregnant women were exposed to the programme. | Pre post | ^182^ | 3 |  |  |
| Community-based distribution of misoprostol to persons in the community, to TBAs, and to drug keepers for prevention of postpartum hemorrhage (PPH) in Nigeria | Community leaders and selected community members participated in a series of dialogs. Additionally, community education, information and dramas sessions were held. Twenty nine community oriented resource persons (CORPs), 27 drug keepers and 41 traditional birth attendants (TBAs) were involved in the intervention. Women identified TBAs and CORPs as the single most important source of information about misoprostol. | Cross sectional | ^183^ | 3 | Community mobilization might facilitate the uptake of public health interventions such as community-based distribution of misoprostol to prevent PPH, undertaken in five communities around Zaria, Nigeria. | Theories of community participation to address MNH issues to be assessed in other settings. Process and impact evaluations not yet published in this study. |
| Linking community and facility-based services of MNH care in Matlab, Bangladesh | In the intervention areas, coverage of ANC, facility delivery, rates of caesarian sections were higher compared to before implementation. PMR decreased over that time period in the intervention area, significantly more so than in the comparison area. | Pre post with control area | ^184^ | 3 | The intervention followed a continuum of care model by improving established links between community- and facility-based services. | Extend of community participation and mobilization unclear. |
| Intervention teaching mothers and their home birth attendants (TBA) in the recognition of danger signs; improvement of transports to EmOC | In rural Uttar Pradesh (UP), India, retention of knowledge and skills for recognition and intervention for maternal bleeding and newborn sepsis was enhanced when pictorial depictions of the problem or take action message or both were used as memory aids. SBR did not change with training. | Pre post | ^185^ | 3 | Community mobilization efforts targeted at reducing delays in transport to EmOC and to increase use of family planning. | Assessment needs better presentation of results. |
| Community participatory birth preparedness using visual aids | Birth preparedness interventions should not only address women, but the community at large who supports pregnant women. Over the year in which the project was undertaken, there was a 22% increase in antenatal care, a 32% increase in the number of women delivered by a midwife, and a 281% increase in referrals to hospital. | Qualitative | ^186^ | C | Communities that are poor and isolated are responsive to the health needs of their women as they give birth, and articulate their needs when given the opportunity. | Needs health impact assessment related to the intervention. |
| Positive deviance approach to improve newborn care | A positive deviance (PD) inquiry identifies uncommon, model practices from outliers, that a follow-on programme can facilitate to implement in its activities. The use of qualitative methods can help identify positive deviants to mobilize communities to improve newborn care. | Qualitative | ^187^ | B | Conducted in 2 communities (total population about 5,000 each) in two project areas in Haripur District, Pakistan among Afghan refugees and among local Pakistanis. | PD has been used for infant nutrition programmes, need to assess in MNH programmes. |

**F. Leadership and governance**

| **Innovative MNH approach** | **Summary of Evidence** | | | |
| --- | --- | --- | --- | --- |
|  | **Evidence** | **Type of Study** | **Reference** | **Grade** |
| **Political leadership and governance** | | | | |
| Public-private sector partnerships to improve maternal health care | A large scale PPP in Gujarat (Chiranjeevi) connected 800 OB providers to provide health care to poor women and increased the proportion of women delivering at health facilities from 27 to 53%. (also see table “Financing”) | Pre post | ^84^ | 3 |
|  | Private providers (nurses, midwives) can contribute to maternal care in low-income settings, but -as in the public sector- will need improvements in the health system (such as higher-level referral facilities) to address constraints similar to the ones encountered by public sector providers. | Narrative review | ^188^ | 4 |
|  | There is limited evidence on whether PPP have contributed to improving access to and affordability of MNH services. | Narrative review | ^189^ | 4 |
| Health systems reform to improve maternal health care in the Philippines | Maternal health systems reform increased facility-based deliveries and had positive synergistic effects on workforce and financing. MMR declined more in reform areas than in comparison areas in that time period. | Pre post with comparison areas | ^190^ | 3 |
| Use of research and policies to develop community-based newborn care package | An expert group in Nepal reviewed existing evidence, developed a prioritization tool and conducted learning visits to design the first national newborn health policy in South Asia, Nepal's Community-Based Newborn Care Package, which is delivered through nationally available cadres of Female Community Health Volunteers. | Policy Analysis | ^191^ | 4 |
| Integration of skilled birth attendance into National Master Plan for Action policy | In Vietnam, civil society organizations contributed to the policy making processes for a policy on skilled attendance at birth. | Case study | ^192^ | 4 |
| Use of evidence, influence of local high-profile champions to improve maternal iodine supplementation in Thailand. | Rapid provision of evidence, such as lit reviews and cross-sectional studies, can be efficient in influencing MNH policy making, as shown in the case of maternal iodine supplementation in Thailand. | Case study | ^193^ | 4 |
| IMDA (investigating maternal death and act) approach: Data sharing of maternal deaths audits to develop recommendations for action in Zambia | Qualitative investigation of maternal mortality can help key decision makers draw recommendations to be implemented to improve maternal health. (Qualitative methods were used to develop, not evaluate, the intervention. The intervention consisted in application of qualitative methods (which are not well described) to draw recommendations.) | Case study | ^194^ | B |
| Political commitment in Sri Lanka to provide free maternal health care | The relatively well-developed maternal care system in Sri Lanka reflects political commitment to provide free maternal health care. | Case study | ^195^ | 4 |
| Use of evidence, influence of local high-profile champions to improve newborn health | Locally generated evidence and global evidence (the Lancet series on newborn health) and high-profile champions, together with community initiatives and intensive donor funding, helped influence policy to improve newborn survival in Bangladesh. | Policy analysis | ^196^ | 4 |
| Use of data and effective national partnerships for advocacy and planning of a national newborn strategy in Nepal | Political commitment supported reduction in fertility, improvements in female education and promotion of skilled birth attendance, as well as increased coverage of community-based child health interventions. Through strategic use of global and national data and effective partnerships using primarily a selective newborn-focused approach for advocacy and planning, Nepal was the first low-income country to create a national newborn strategy, the Community-Based Newborn Care Package, piloted in 10 of 75 districts, with plans to increase to 35 districts in mid-2013. The policy influenced similar strategies in other countries. | Policy analysis | ^166^ | 4 |
| Approaches such as home visits by CHW, IMNCI programmes, improve infrastructure and workforce support in South East Asia | Innovative neonatal health schemes unified in a concerted health systems strengthening effort rather than in a multitude of programmes are cost-effective and can be scaled-up rapidly. | Narrative Review | ^197^ | 4 |
| Integration of newborn care into existing community-based packages: national MNCH Programme in Pakistan | In the time period of implementation of newborn care interventions by the Lady Health Worker programme, NMR declined less in Pakistan than the global average. The national MNCH Programme catalyzed newborn services at both facility and community levels. Civil society and academics have linked with government and several research studies have been highly influential. Since 2005, donor funding mentioning the term 'newborn' has increased more for Pakistan than for other countries. | Policy analysis | ^198^ | 4 |
| Establishment of Ethiopia's health extension programme to improve MH services delivery in remote areas | The Health Extension Program was initiated as part of the Health Sector Development Program in Ethiopia to improve MMR and other indicators (such as skilled birth attendance), which remain far from MDGs. | Case study | ^199^ | 4 |
| Implementation of a community-based MNH package through an expert platform to raise high-level attention for comprehensive policy change in Malawi. | Through initial entry initiatives at the facility level (such as kangaroo care), policy transition towards integrated approaches and community-based maternal and newborn care packages. | Policy analysis | ^200^ | 4 |
| Improving access, coverage, and quality of MNH care in Brazil through rights-based programming | Right-based approaches are new to maternal care in Brazil, might help restructure MNH care towards a more humanistic care model and provide a framework for system evaluation. | Case study | ^201^ | 4 |
| National policy to exempt fee for delivery care in Ghana | Existing evaluation studies suggest that the fee exemption policy in Ghana benefitted the poors' access to maternity care. Adequate funding and strong institutional ownership are essential to ensure the sustainability of the policy and high quality standards of care. | Policy analysis (also see table “Financial approaches”) | ^149^ | 4 |
| Using a micro-planning strategy (reaching every district RED) to improve access to MNH services | Barrier analysis and mapping approaches helped problem solving at the local level to reach remote populations in Mongolia. | Case study | ^202^ | 4 |
| Development of a nation-specific situation analysis and action plan for newborn health in Nigeria | Policy and guidelines need to include MNH and need to address IMNCI implementation, adequate funding and political stewardship, and the planning for CHW to bridge shortages in workforce | Report | ^203^ | 4 |
| Community mobilization to improve transport and access to maternal health services | Community participation programmes in Mexico emphasize support from mothers' networks as well as from government housing and transportation programmes. (also see table “Community participation”) | Qualitative study | ^204^ | B |
| Use of data and effective international partnerships to develop an action plan for MNH in Mesoamerica | A situational analysis of MNH in the region led to specific strategic recommendations emphasizing EmOC and emergency neonatal care, and skilled birth attendance. | Policy analysis | ^205^ | 4 |
| Inter-agency task team initiated Joint Technical Missions to galvanize country action for PMTCT scale-up | Joint technical missions informing policy and programme decisions identified the critical components of successful national scale-up of PMTCT programmes in Africa and Asia. | Case study | ^206^ | 4 |
| Engaging NGOs in in collaborative HIV/TB activities to strengthen PMTCT | Engaging CHW and NGOs working on TB/HIV in PMTCT activities might help improve HIV prevention in South Africa. | Case study | ^207^ | A |
| Regional multilateral partnership (network between 4 countries) to improve childbirth practices in Arab countries | The "Choices and Challenges in Changing Childbirth" research network has conducted research that aims to influence practices with regionally relevant high-quality evidence. | Case study | ^208^ | 4 |

**References**

**1.** Srofenyoh E, Ivester T, Engmann C, Olufolabi A, Bookman L, Owen M. Advancing obstetric and neonatal care in a regional hospital in Ghana via continuous quality improvement. *Int J Gynaecol Obstet.* 2012;116(1):17-21.

**2.** Igwegbe AO, Eleje GU, Ugboaja JO, Ofiaeli RO. Improving maternal mortality at a university teaching hospital in Nnewi, Nigeria. *Int J Gynaecol Obstet.* 2012;116(3):197-200.

**3.** Mansour M, Mansour JB, El Swesy AH. Scaling up proven public health interventions through a locally owned and sustained leadership development programme in rural Upper Egypt. *Hum Resour Health.* 2010;8(1):1.

**4.** Spector JM, Agrawal P, Kodkany B, Lipsitz S, Lashoher A, Dziekan G, Bahl R, Merialdi M, Mathai M, Lemer C, Gawande A. Improving quality of care for maternal and newborn health: prospective pilot study of the WHO safe childbirth checklist program. *PLoS ONE.* 2012;7(5):e35151.

**5.** Aghlmand S, Akbari F, Lameei A, Mohammad K, Small R, Arab M. Developing evidence-based maternity care in Iran: a quality improvement study. *BMC Pregnancy Childbirth.* 2008;8:20.

**6.** USAID. The Role of Modern Quality Improvement in Enhancing Maternal, Newborn, and Child Health Programs. 2012;acessed at <http://www.hciproject.org/sites/default/files/Role%20of%20QI%20in%20Enhancing%20MNCH%20Programs_June2012_0.pdf>.

**7.** Padmanaban P, Raman PS, Mavalankar DV. Innovations and challenges in reducing maternal mortality in Tamil Nadu, India. *J Health Popul Nutr.* 2009;27(2):202-219.

**8.** Zhou H, Zhao CX, Wang XL, Xv YC, Shi L, Wang Y. Effectiveness of an intervention on uptake of maternal care in four counties in Ningxia, China. *Trop Med Int Health.* 2012.

**9.** Warren C, Mwangi A, Oweya E, Kamunya R, Koskei N. Safeguarding maternal and newborn health: improving the quality of postnatal care in Kenya. *Int J Qual Health Care.* 2010;22(1):24-30.

**10.** Liu X, Yan H, Wang D. The evaluation of "Safe Motherhood" program on maternal care utilization in rural western China: a difference in difference approach. *BMC Public Health.* 2010;10(566):(22 September 2010).

**11.** Neogi SB, Malhotra S, Zodpey S, Mohan P. Challenges in scaling up of special care newborn units--lessons from India. *Indian Pediatr.* 2012;48(12):931-935.

**12.** Landre-Peigne C, Ka AS, Peigne V, Bougere J, Seye MN, Imbert P. Efficacy of an infection control programme in reducing nosocomial bloodstream infections in a Senegalese neonatal unit. *J Hosp Infect.* 2011;79(2):161-165.

**13.** Darmstadt GL, Nawshad Uddin Ahmed AS, Saha SK, Azad Chowdhury MA, Alam MA, Khatun M, Black RE, Santosham M. Infection control practices reduce nosocomial infections and mortality in preterm infants in Bangladesh. *J Perinatol.* 2005;25(5):331-335.

**14.** Carvalho DS, Novaes HM. [Evaluation of the prenatal care program in Curitiba, Parana, Brazil: a cohort study of primigravidae]. *Cad Saude Publica.* 2004;20 Suppl 2:S220-230.

**15.** Honikman S, van Heyningen T, Field S, Baron E, Tomlinson M. Stepped care for maternal mental health: a case study of the perinatal mental health project in South Africa. *PLoS Med.* 2012;9(5):e1001222.

**16.** Liang J, Li X, Dai L, Zeng W, Li Q, Li M, Zhou R, He C, Wang Y, Zhu J. The changes in maternal mortality in 1000 counties in mid-Western China by a government-initiated intervention. *PLoS ONE.* 2012;7(5):e37458.

**17.** Clapham S, Basnet I, Pathak LR, McCall M. The evolution of a quality of care approach for improving emergency obstetric care in rural hospitals in Nepal. *Int J Gynaecol Obstet.* 2004;86(1):86-97; discussion 85.

**18.** Sloan NL, Ahmed S, Mitra SN, Choudhury N, Chowdhury M, Rob U, Winikoff B. Community-based kangaroo mother care to prevent neonatal and infant mortality: a randomized, controlled cluster trial. *Pediatrics.* 2008;121(5):e1047-1059.

**19.** Ahmed S, Mitra SN, Chowdhury AM, Camacho LL, Winikoff B, Sloan NL. Community Kangaroo Mother Care: implementation and potential for neonatal survival and health in very low-income settings. *J Perinatol.* 2011;31(5):361-367.

**20.** Bergh AM, Arsalo I, Malan AF, Patrick M, Pattinson RC, Phillips N. Measuring implementation progress in kangaroo mother care. *Acta Paediatr.* 2005;94(8):1102-1108.

**21.** Bergh AM, Pattinson RC. Development of a conceptual tool for the implementation of kangaroo mother care. *Acta Paediatr.* 2003;92(6):709-714.

**22.** Bergh AM, Manu R, Davy K, van Rooyen E, Asare GQ, Williams JK, Dedzo M, Twumasi A, Nang-Beifubah A. Translating research findings into practice--the implementation of kangaroo mother care in Ghana. *Implement Sci.* 2012;7:75.

**23.** Gontijo TL, Xavier CC, Freitas MI. [Evaluation of the implementation of Kangaroo Care by health administrators, professionals, and mothers of newborn infants]. *Cad Saude Publica.* 2012;28(5):935-944.

**24.** Pattinson RC, Arsalo I, Bergh AM, Malan AF, Patrick M, Phillips N. Implementation of kangaroo mother care: a randomized trial of two outreach strategies. *Acta Paediatr.* 2005;94(7):924-927.

**25.** Bergh AM, van Rooyen E, Pattinson RC. Scaling up kangaroo mother care in South Africa: 'on-site' versus 'off-site' educational facilitation. *Hum Resour Health.* 2008;6:13.

**26.** Bhatnagar S, Wadhwa N, Aneja S, Lodha R, Kabra SK, Natchu UC, Sommerfelt H, Dutta AK, Chandra J, Rath B, Sharma M, Sharma VK, Kumari M, Strand TA. Zinc as adjunct treatment in infants aged between 7 and 120 days with probable serious bacterial infection: a randomised, double-blind, placebo-controlled trial. *Lancet.* 2012;379(9831):2072-2078.

**27.** da Silva CL, Saunders C, Szarfarc SC, Fujimori E, da Veiga GV. Anaemia in pregnant women before and after the mandatory fortification of wheat and corn flours with iron. *Public Health Nutr.* 2012;15(10):1802-1809.

**28.** Frith AL, Naved RT, Persson LA, Rasmussen KM, Frongillo EA. Early participation in a prenatal food supplementation program ameliorates the negative association of food insecurity with quality of maternal-infant interaction. *J Nutr.* 2012;142(6):1095-1101.

**29.** Jarjou LM, Prentice A, Sawo Y, Laskey MA, Bennett J, Goldberg GR, Cole TJ. Randomized, placebo-controlled, calcium supplementation study in pregnant Gambian women: effects on breast-milk calcium concentrations and infant birth weight, growth, and bone mineral accretion in the first year of life. *Am J Clin Nutr.* 2006;83(3):657-666.

**30.** Persson LA, Arifeen S, Ekstrom EC, Rasmussen KM, Frongillo EA, Yunus M. Effects of prenatal micronutrient and early food supplementation on maternal hemoglobin, birth weight, and infant mortality among children in Bangladesh: the MINIMat randomized trial. *JAMA.* 2012;307(19):2050-2059.

**31.** Ahrari M, Houser RF, Yassin S, Mogheez M, Hussaini Y, Crump P, Darmstadt GL, Marsh D, Levinson FJ. A positive deviance-based antenatal nutrition project improves birth-weight in Upper Egypt. *J Health Popul Nutr.* 2006;24(4):498-507.

**32.** Nahar S, Mascie-Taylor CG, Begum HA. Impact of targeted food supplementation on pregnancy weight gain and birth weight in rural Bangladesh: an assessment of the Bangladesh Integrated Nutrition Program (BINP). *Public Health Nutr.* 2009;12(8):1205-1212.

**33.** Perez-Escamilla R, Curry L, Minhas D, Taylor L, Bradley E. Scaling up of breastfeeding promotion programs in low- and middle-income countries: the "breastfeeding gear" model. *Adv Nutr.* 2012;3(6):790-800.

**34.** Bashour HN, Kharouf MH, Abdulsalam AA, El Asmar K, Tabbaa MA, Cheikha SA. Effect of postnatal home visits on maternal/infant outcomes in Syria: a randomized controlled trial. *Public Health Nurs.* 2008;25(2):115-125.

**35.** Chola L, Nkonki L, Kankasa C, Nankunda J, Tumwine J, Tylleskar T, Robberstad B. Cost of individual peer counselling for the promotion of exclusive breastfeeding in Uganda. *Cost Eff Resour Alloc.* 2011;9(1):11.

**36.** Qureshi AM, Oche OM, Sadiq UA, Kabiru S. Using community volunteers to promote exclusive breastfeeding in Sokoto State, Nigeria. *Pan Afr Med J.* 2011;10:8.

**37.** Bhandari N, Kabir AK, Salam MA. Mainstreaming nutrition into maternal and child health programmes: scaling up of exclusive breastfeeding. *Matern Child Nutr.* 2008;4 Suppl 1:5-23.

**38.** Eckermann E, Deodato G. Maternity waiting homes in Southern Lao PDR: the unique 'silk home'. *J Obstet Gynaecol Res.* 2008;34(5):767-775.

**39.** Wild K, Barclay L, Kelly P, Martins N. The tyranny of distance: maternity waiting homes and access to birthing facilities in rural Timor-Leste. *Bull World Health Organ.* 2012;90(2):97-103.

**40.** Garcia Prado A, Cortez R. Maternity waiting homes and institutional birth in Nicaragua: policy options and strategic implications. *Int J Health Plann Manage.* 2011;27(2):150-166.

**41.** Rakhshani A, Nagarathna R, Mhaskar R, Mhaskar A, Thomas A, Gunasheela S. The effects of yoga in prevention of pregnancy complications in high-risk pregnancies: A randomized controlled trial. *Prev Med.* 2012;55(4):333-340.

**42.** Gao LL, Chan SW, Sun K. Effects of an interpersonal-psychotherapy-oriented childbirth education programme for Chinese first-time childbearing women at 3-month follow up: randomised controlled trial. *Int J Nurs Stud.* 2011;49(3):274-281.

**43.** Jafari F, Eftekhar H, Mohammad K, Fotouhi A. Does group prenatal care affect satisfaction and prenatal care utilization in Iranian pregnant women? *Iranian Journal of Public Health.* 2010;39(2):52-62.

**44.** Kausar F, Morris JL, Fathalla M, Ojengbede O, Fabamwo A, Mourad-Youssif M, Morhason-Bello IO, Galadanci H, Nsima D, Butrick E, Miller S. Nurses in low resource settings save mothers' lives with non-pneumatic anti-shock garment. *MCN Am J Matern Child Nurs.* 2012;37(5):308-316.

**45.** Miller S, Turan JM, Dau K, Fathalla M, Mourad M, Sutherland T, Hamza S, Lester F, Gibson EB, Gipson R, Nada K, Hensleigh P. Use of the non-pneumatic anti-shock garment (NASG) to reduce blood loss and time to recovery from shock for women with obstetric haemorrhage in Egypt. *Glob Public Health.* 2007;2(2):110-124.

**46.** Miller S, Lester F, Hensleigh P. Prevention and treatment of postpartum hemorrhage: new advances for low-resource settings. *J Midwifery Womens Health.* 2004;49(4):283-292.

**47.** Baker EC, Hezelgrave N, Magesa SM, Edmonds S, de Greeff A, Shennan A. Introduction of automated blood pressure devices intended for a low resource setting in rural Tanzania. *Trop Doct.* 2012;42(2):101-103.

**48.** Ouma MN, Chemwolo BT, Pastakia S, Christoffersen-Deb A, Washington S. Pilot study of single-use obstetric emergency medical kits to reduce maternal mortality. *Int J Gynaecol Obstet.* 2012;119(1):49-52.

**49.** Malkin R, Howard C. A Foilized Polyethylene Pouch for the Prevention of Transmission of HIV from Mother to Child. *Open Biomed Eng J.* 2012;6:92-97.

**50.** Hofmeyr GJ, Haws RA, Bergstrom S, Lee AC, Okong P, Darmstadt GL, Mullany LC, Oo EK, Lawn JE. Obstetric care in low-resource settings: what, who, and how to overcome challenges to scale up? *Int J Gynaecol Obstet.* 2009;107 Suppl 1:S21-44, S44-25.

**51.** Thairu L. Medical devices for pregnancy and childbirth in the developing world. *Health and Technology.* 2012;2:13.

**52.** Hundley VA, Avan BI, Braunholtz D, Graham WJ. Are birth kits a good idea? A systematic review of the evidence. *Midwifery.* 2012;28(2):204-215.

**53.** Hundley VA, Avan BI, Braunholtz D, Fitzmaurice AE, Graham WJ. Lessons regarding the use of birth kits in low resource countries. *Midwifery.* 2011;27(6):e222-230.

**54.** Howitt P, Darzi A, Yang GZ, Ashrafian H, Atun R, Barlow J, Blakemore A, Bull AM, Car J, Conteh L, Cooke GS, Ford N, Gregson SA, Kerr K, King D, Kulendran M, Malkin RA, Majeed A, Matlin S, Merrifield R, Penfold HA, Reid SD, Smith PC, Stevens MM, Templeton MR, Vincent C, Wilson E. Technologies for global health. *Lancet.* 2012;380(9840):507-535.

**55.** Goudar SS, Somannavar MS, Clark R, Lockyer JM, Revankar AP, Fidler HM, Sloan NL, Niermeyer S, Keenan WJ, Singhal N. Stillbirth and newborn mortality in India after helping babies breathe training. *Pediatrics.* 2013;131(2):e344-352.

**56.** Msemo G, Massawe A, Mmbando D, Rusibamayila N, Manji K, Kidanto HL, Mwizamuholya D, Ringia P, Ersdal HL, Perlman J. Newborn mortality and fresh stillbirth rates in Tanzania after helping babies breathe training. *Pediatrics.* 2013;131(2):e353-360.

**57.** Thairu L, Wirth M, Lunze K. Innovative newborn health technology for resource-limited environments. *Trop Med Int Health.* 2013;18(1):117-128.

**58.** Blencowe H, Cousens S, Mullany LC, Lee AC, Kerber K, Wall S, Darmstadt GL, Lawn JE. Clean birth and postnatal care practices to reduce neonatal deaths from sepsis and tetanus: a systematic review and Delphi estimation of mortality effect. *BMC Public Health.* 2011;11 Suppl 3:S11.

**59.** Mullany LC, Saha SK, Shah R, Islam MS, Rahman M, Islam M, Talukder RR, El Arifeen S, Darmstadt GL, Baqui AH. Impact of 4.0% chlorhexidine cord cleansing on the bacteriologic profile of the newborn umbilical stump in rural Sylhet District, Bangladesh: a community-based, cluster-randomized trial. *Pediatr Infect Dis J.* 2012;31(5):444-450.

**60.** Gray PH, Flenady V. Cot-nursing versus incubator care for preterm infants. *Cochrane Database Syst Rev.* 2011(8):CD003062.

**61.** PATH. Newborn Thermal Care Devices - Establishing a Value Proposition for Low-Resource Settings. 2009;accessed online at <http://www.path.org/publications/files/TS_newborn_thermal_rpt.pdf>.

**62.** Duke T, Subhi R, Peel D, Frey B. Pulse oximetry: technology to reduce child mortality in developing countries. *Ann Trop Paediatr.* 2009;29(3):165-175.

**63.** Bhutani VK, Cline BK, Donaldson KM, Vreman HJ. The need to implement effective phototherapy in resource-constrained settings. *Semin Perinatol.* 2011;35(3):192-197.

**64.** Thukral A, Sasi A, Chawla D, Datta P, Wahid S, Rao S, Kannan V, Veeragandam A, Murki S, Deorari AK. Online Neonatal Training and Orientation Programme in India (ONTOP-IN)--The Way Forward for Distance Education in Developing Countries. *J Trop Pediatr.* 2012.

**65.** Deorari A, Thukral A, Aruna V. Online learning in newborn health: a distance learning model. *Natl Med J India.* 2012;25(1):31-32.

**66.** Woods D, Attwell A, Ross K, Theron G. Text messages as a learning tool for midwives. *S Afr Med J.* 2012;102(2):100-101.

**67.** Olson KR, Caldwell A, Sihombing M, Guarino AJ, Nelson BD. Community-based newborn resuscitation among frontline providers in a low-resource country. *Int J Gynaecol Obstet.* 2012;119(3):244-247.

**68.** Brown H, Hofmeyr GJ, Nikodem VC, Smith H, Garner P. Promoting childbirth companions in South Africa: a randomised pilot study. *BMC Med.* 2007;5:7.

**69.** Lewin S, Munabi-Babigumira S, Glenton C, Daniels K, Bosch-Capblanch X, van Wyk BE, Odgaard-Jensen J, Johansen M, Aja GN, Zwarenstein M, Scheel IB. Lay health workers in primary and community health care for maternal and child health and the management of infectious diseases. *Cochrane Database Syst Rev.* 2010(3):CD004015.

**70.** Gogia S, Ramji S, Gupta P, Gera T, Shah D, Mathew JL, Mohan P, Panda R. Community based newborn care: a systematic review and metaanalysis of evidence: UNICEF-PHFI series on newborn and child health, India. *Indian Pediatr.* 2011;48(7):537-546.

**71.** Hafeez A, Mohamud BK, Shiekh MR, Shah SA, Jooma R. Lady health workers programme in Pakistan: challenges, achievements and the way forward. *J Pak Med Assoc.* 2011;61(3):210-215.

**72.** Shewade HD, Aggarwal AK, Bharti B. Integrated Management of Neonatal and Childhood Illness (IMNCI): Skill Assessment of Health and Integrated Child Development Scheme (ICDS) Workers to Classify Sick Under-five Children. *Indian J Pediatr.* 2012.

**73.** Khanal S, Sharma J, Gc VS, Dawson P, Houston R, Khadka N, Yengden B. Community health workers can identify and manage possible infections in neonates and young infants: MINI--a model from Nepal. *J Health Popul Nutr.* 2011;29(3):255-264.

**74.** Dynes M, Buffington ST, Carpenter M, Handley A, Kelley M, Tadesse L, Beyene HT, Sibley L. Strengthening maternal and newborn health in rural Ethiopia: Early results from frontline health worker community maternal and newborn health training. *Midwifery.* 2012.

**75.** Medhanyie A, Spigt M, Kifle Y, Schaay N, Sanders D, Blanco R, Geertjan D, Berhane Y. The role of health extension workers in improving utilization of maternal health services in rural areas in Ethiopia: a cross sectional study. *BMC Health Serv Res.* 2012;12:352.

**76.** Nelson BD, Ahn R, Fehling M, Eckardt MJ, Conn KL, El-Bashir A, Tiernan M, Purcell G, Burke TF. Evaluation of a novel training package among frontline maternal, newborn, and child health workers in South Sudan. *Int J Gynaecol Obstet.* 2012;119(2):130-135.

**77.** Kadomoto N, Iwasa H, Takahashi M, Dulnuan MM, Kai I. Ifugao males, learning and teaching for the improvement of maternal and child health status in the Philippines: an evaluation of a program. *BMC Public Health.* 2011;11:280.

**78.** Kim MH, Ahmed S, Buck WC, Preidis GA, Hosseinipour MC, Bhalakia A, Nanthuru D, Kazembe PN, Chimbwandira F, Giordano TP, Chiao EY, Schutze GE, Kline MW. The Tingathe programme: a pilot intervention using community health workers to create a continuum of care in the prevention of mother to child transmission of HIV (PMTCT) cascade of services in Malawi. *J Int AIDS Soc.* 2012;15 Suppl 2:17389.

**79.** Chanda P, Hamainza B, Moonga HB, Chalwe V, Banda P, Pagnoni F. Relative costs and effectiveness of treating uncomplicated malaria in two rural districts in Zambia: implications for nationwide scale-up of home-based management. *Malar J.* 2011;10:159.

**80.** Vieira C, Portela A, Miller T, Coast E, Leone T, Marston C. Increasing the use of skilled health personnel where traditional birth attendants were providers of childbirth care: a systematic review. *PLoS ONE.* 2012;7(10):e47946.

**81.** Wilson A, Lissauer D, Thangaratinam S, Khan KS, MacArthur C, Coomarasamy A. A comparison of clinical officers with medical doctors on outcomes of caesarean section in the developing world: meta-analysis of controlled studies. *BMJ.* 2011;342:d2600.

**82.** Nyamtema AS, Pemba SK, Mbaruku G, Rutasha FD, van Roosmalen J. Tanzanian lessons in using non-physician clinicians to scale up comprehensive emergency obstetric care in remote and rural areas. *Hum Resour Health.* 2011;9:28.

**83.** Pereira C, Mbaruku G, Nzabuhakwa C, Bergstrom S, McCord C. Emergency obstetric surgery by non-physician clinicians in Tanzania. *Int J Gynaecol Obstet.* 2011;114(2):180-183.

**84.** Mavalankar D, Singh A, Patel SR, Desai A, Singh PV. Saving mothers and newborns through an innovative partnership with private sector obstetricians: Chiranjeevi scheme of Gujarat, India. *Int J Gynaecol Obstet.* 2009;107(3):271-276.

**85.** Sen A, Mahalanabis D, Singh AK, Som TK, Bandyopadhyay S, Roy S. Newborn Aides: an innovative approach in sick newborn care at a district-level special care unit. *J Health Popul Nutr.* 2007;25(4):495-501.

**86.** Jennings L, Yebadokpo AS, Affo J, Agbogbe M. Antenatal counseling in maternal and newborn care: use of job aids to improve health worker performance and maternal understanding in Benin. *BMC Pregnancy Childbirth.* 2010;10:75.

**87.** Jennings L, Yebadokpo AS, Affo J, Agbogbe M, Tankoano A. Task shifting in maternal and newborn care: a non-inferiority study examining delegation of antenatal counseling to lay nurse aides supported by job aids in Benin. *Implement Sci.* 2011;6:2.

**88.** USAID. IMPROVING MATERNAL AND NEWBORN CARE COUNSELING IN BENIN: OPERATIONS RESEARCH ON USE OF JOB AIDS AND TASK SHIFTING. 2009.

**89.** Walker DM, Cohen SR, Estrada F, Monterroso ME, Jenny A, Fritz J, Fahey JO. PRONTO training for obstetric and neonatal emergencies in Mexico. *Int J Gynaecol Obstet.* 2011;116(2):128-133.

**90.** Wall SN, Lee AC, Carlo W, Goldenberg R, Niermeyer S, Darmstadt GL, Keenan W, Bhutta ZA, Perlman J, Lawn JE. Reducing intrapartum-related neonatal deaths in low- and middle-income countries-what works? *Semin Perinatol.* 2010;34(6):395-407.

**91.** USAID. Interventions for Impact in Essential Obstetric and Newborn Care. 2011a;acessed at <http://www.mchip.net/sites/default/files/mchipfiles/FINAL%20AddisMeetingReport_0627.pdf>

**92.** Uxa F, Bacci A, Mangiaterra V, Chiaffoni GP. Essential newborn care training activities: 8 years of experience in Eastern European, Caucasian and Central Asian countries. *Semin Fetal Neonatal Med.* 2006;11(1):58-64.

**93.** Carlo WA, Goudar SS, Jehan I, Chomba E, Tshefu A, Garces A, Parida S, Althabe F, McClure EM, Derman RJ, Goldenberg RL, Bose C, Krebs NF, Panigrahi P, Buekens P, Chakraborty H, Hartwell TD, Wright LL. Newborn-care training and perinatal mortality in developing countries. *N Engl J Med.* 2010;362(7):614-623.

**94.** Manasyan A, Chomba E, McClure EM, Wright LL, Krzywanski S, Carlo WA. Cost-effectiveness of essential newborn care training in urban first-level facilities. *Pediatrics.* 2011;127(5):e1176-1181.

**95.** McClure EM, Carlo WA, Wright LL, Chomba E, Uxa F, Lincetto O, Bann C. Evaluation of the educational impact of the WHO Essential Newborn Care course in Zambia. *Acta Paediatr.* 2007;96(8):1135-1138.

**96.** Chomba E, McClure EM, Wright LL, Carlo WA, Chakraborty H, Harris H. Effect of WHO newborn care training on neonatal mortality by education. *Ambul Pediatr.* 2008;8(5):300-304.

**97.** Xu T, Wang HS, Ye HM, Yu RJ, Huang XH, Wang DH, Wang LX, Feng Q, Gong LM, Ma Y, Keenan W, Niermeyer S. Impact of a nationwide training program for neonatal resuscitation in China. *Chin Med J (Engl).* 2012;125(8):1448-1456.

**98.** Lalonde AB, McMullen H. A report on the FIGO Saving Mothers and Newborns Project. *J Obstet Gynaecol Can.* 2009;31(10):970-973.

**99.** Singhal N, Lockyer J, Fidler H, Aziz K, McMillan D, Qiu X, Ma X, Du L, Lee SK. Acute Care of At-Risk Newborns (ACoRN): quantitative and qualitative educational evaluation of the program in a region of China. *BMC Med Educ.* 2012;12:44.

**100.** Kumar D, Aggarwal AK, Kumar R. The effect of interrupted 5-day training on Integrated Management of Neonatal and Childhood Illness on the knowledge and skills of primary health care workers. *Health Policy Plan.* 2009;24(2):94-100.

**101.** Mohan P, Kishore B, Singh S, Bahl R, Puri A, Kumar R. Assessment of implementation of integrated management of neonatal and childhood illness in India. *J Health Popul Nutr.* 2012;29(6):629-638.

**102.** Darlow BA, Zin AA, Beecroft G, Moreira ME, Gilbert CE. Capacity building of nurses providing neonatal care in Rio de Janeiro, Brazil: methods for the POINTS of care project to enhance nursing education and reduce adverse neonatal outcomes. *BMC Nurs.* 2012;11:3.

**103.** Senarath U, Fernando DN, Rodrigo I. Effect of training for care providers on practice of essential newborn care in hospitals in Sri Lanka. *J Obstet Gynecol Neonatal Nurs.* 2007;36(6):531-541.

**104.** Osorno LR, Campos MC, Cook LJ, Vela GR, Davila JR. Effectiveness of a regional self-study perinatal education programme: a successful adaptation in Yucatan, Mexico. *Med Educ.* 2006;40(8):816-823.

**105.** van Lonkhuijzen L, Dijkman A, van Roosmalen J, Zeeman G, Scherpbier A. A systematic review of the effectiveness of training in emergency obstetric care in low-resource environments. *BJOG.* 2010;117(7):777-787.

**106.** Islam MT, Haque YA, Waxman R, Bhuiyan AB. Implementation of emergency obstetric care training in Bangladesh: lessons learned. *Reprod Health Matters.* 2006;14(27):61-72.

**107.** Evans CL, Maine D, McCloskey L, Feeley FG, Sanghvi H. Where there is no obstetrician--increasing capacity for emergency obstetric care in rural India: an evaluation of a pilot program to train general doctors. *Int J Gynaecol Obstet.* 2009;107(3):277-282.

**108.** Foster J, Regueira Y, Burgos RI, Sanchez AH. Midwifery curriculum for auxiliary maternity nurses: a case study in the Dominican Republic. *J Midwifery Womens Health.* 2005;50(4):e45-49.

**109.** Holzgreve W, Greiner D, Schwidtal P. Maternal mortality in Eritrea: Improvements associated with centralization of obstetric services. *Int J Gynaecol Obstet.* 2012;119 Suppl 1:S50-54.

**110.** Hafeez A, Zafar S, Qureshi F, Mirza I, Bile K, Southall DP. Emergency maternal and child health training courses and advocacy to achieve millennium development goals in a poorly resourced country; challenges and opportunities. *J Pak Med Assoc.* 2009;59(4):243-246.

**111.** Ohnishi M, Nakamura K, Takano T. Training of healthcare personnel to improve performance of community-based antenatal care program. *Adv Health Sci Educ Theory Pract.* 2007;12(2):147-156.

**112.** Wilson A, Gallos ID, Plana N, Lissauer D, Khan KS, Zamora J, MacArthur C, Coomarasamy A. Effectiveness of strategies incorporating training and support of traditional birth attendants on perinatal and maternal mortality: meta-analysis. *BMJ.* 2011;343:d7102.

**113.** Sibley LM, Sipe TA, Barry D. Traditional birth attendant training for improving health behaviours and pregnancy outcomes. *Cochrane Database Syst Rev.* 2012;8:CD005460.

**114.** Gill CJ, Phiri-Mazala G, Guerina NG, Kasimba J, Mulenga C, MacLeod WB, Waitolo N, Knapp AB, Mirochnick M, Mazimba A, Fox MP, Sabin L, Seidenberg P, Simon JL, Hamer DH. Effect of training traditional birth attendants on neonatal mortality (Lufwanyama Neonatal Survival Project): randomised controlled study. *BMJ.* 2011;342:d346.

**115.** Garces A, McClure EM, Hambidge M, Krebs NF, Mazariegos M, Wright LL, Moore J, Carlo WA. Training traditional birth attendants on the WHO Essential Newborn Care reduces perinatal mortality. *Acta Obstet Gynecol Scand.* 2012;91(5):593-597.

**116.** Matendo R, Engmann C, Ditekemena J, Gado J, Tshefu A, Kinoshita R, McClure EM, Moore J, Wallace D, Carlo WA, Wright LL, Bose C. Reduced perinatal mortality following enhanced training of birth attendants in the Democratic Republic of Congo: a time-dependent effect. *BMC Med.* 2011;9:93.

**117.** Miller PC, Rashida G, Tasneem Z, Haque M. The effect of traditional birth attendant training on maternal and neonatal care. *Int J Gynaecol Obstet.* 2012;117(2):148-152.

**118.** Rowen T, Prata N, Passano P. Evaluation of a traditional birth attendant training programme in Bangladesh. *Midwifery.* 2011;27(2):229-236.

**119.** Satishchandra DM, Naik VA, Wantamutte AS, Mallapur MD. Impact of training of traditional birth attendants on the newborn care. *Indian J Pediatr.* 2009;76(1):33-36.

**120.** Satti H, Motsamai S, Chetane P, Marumo L, Barry DJ, Riley J, McLaughlin MM, Seung KJ, Mukherjee JS. Comprehensive approach to improving maternal health and achieving MDG 5: report from the mountains of Lesotho. *PLoS One.* 2012;7(8):e42700.

**121.** Drake JK, Hutchings JE, Elias CJ. Making evidence work for communities: the role of nongovernmental organizations in translating science to programs. *J Womens Health (Larchmt).* 2010;19(11):2119-2124.

**122.** Prata N, Quaiyum MA, Passano P, Bell S, Bohl DD, Hossain S, Azmi AJ, Begum M. Training traditional birth attendants to use misoprostol and an absorbent delivery mat in home births. *Soc Sci Med.* 2012;75(11):2021-2027.

**123.** Jehan K, Sidney K, Smith H, de Costa A. Improving access to maternity services: an overview of cash transfer and voucher schemes in South Asia. *Reprod Health Matters.* 2012;20(39):142-154.

**124.** Gopalan SS, Durairaj V. Addressing maternal healthcare through demand side financial incentives: experience of Janani Suraksha Yojana program in India. *BMC Health Serv Res.* 2012;12:319.

**125.** Sidney K, de Costa A, Diwan V, Mavalankar DV, Smith H. An evaluation of two large scale demand side financing programs for maternal health in India: the MATIND study protocol. *BMC Public Health.* 2012;12:699.

**126.** Ensor T, Clapham S, Prasai DP. What drives health policy formulation: insights from the Nepal maternity incentive scheme? *Health Policy.* 2009;90(2-3):247-253.

**127.** Powell-Jackson T, Hanson K. Financial incentives for maternal health: impact of a national programme in Nepal. *J Health Econ.* 2012;31(1):271-284.

**128.** Basinga P, Gertler PJ, Binagwaho A, Soucat AL, Sturdy J, Vermeersch CM. Effect on maternal and child health services in Rwanda of payment to primary health-care providers for performance: an impact evaluation. *Lancet.* 2011;377(9775):1421-1428.

**129.** Soeters R, Peerenboom PB, Mushagalusa P, Kimanuka C. Performance-based financing experiment improved health care in the Democratic Republic of Congo. *Health Aff (Millwood).* 2011;30(8):1518-1527.

**130.** Ahmed S, Khan MM. A maternal health voucher scheme: what have we learned from the demand-side financing scheme in Bangladesh? *Health Policy Plan.* 2011;26(1):25-32.

**131.** Abuya T, Njuki R, Warren CE, Okal J, Obare F, Kanya L, Askew I, Bellows B. A Policy Analysis of the implementation of a Reproductive Health Vouchers Program in Kenya. *BMC Public Health.* 2012;12:540.

**132.** Agha S. Changes in the proportion of facility-based deliveries and related maternal health services among the poor in rural Jhang, Pakistan: results from a demand-side financing intervention. *Int J Equity Health.* 2011;10:57.

**133.** Agha S. Impact of a maternal health voucher scheme on institutional delivery among low income women in Pakistan. *Reprod Health.* 2011a;8:10.

**134.** Nguyen HT, Hatt L, Islam M, Sloan NL, Chowdhury J, Schmidt JO, Hossain A, Wang H. Encouraging maternal health service utilization: an evaluation of the Bangladesh voucher program. *Soc Sci Med.* 2012;74(7):989-996.

**135.** Bellows B, Kyobutungi C, Mutua MK, Warren C, Ezeh A. Increase in facility-based deliveries associated with a maternal health voucher programme in informal settlements in Nairobi, Kenya. *Health Policy Plan.* 2012;28(2):134-142.

**136.** Bellows B, Warren C, Vonthanak S, Chhorvann C, Sokhom H, Men C, Bajracharya A, Rob U, Rathavy T. Evaluation of the impact of the voucher and accreditation approach on improving reproductive behaviors and status in Cambodia. *BMC Public Health.* 2011;11:667.

**137.** Ahmed S, Khan MM. Is demand-side financing equity enhancing? Lessons from a maternal health voucher scheme in Bangladesh. *Soc Sci Med.* 2011a;72(10):1704-1710.

**138.** Rob U, Rahman M, Bellows B. Evaluation of the impact of the voucher and accreditation approach on improving reproductive behaviors and RH status: Bangladesh. *BMC Public Health.* 2011;11:257.

**139.** Schmidt JO, Ensor T, Hossain A, Khan S. Vouchers as demand side financing instruments for health care: a review of the Bangladesh maternal voucher scheme. *Health Policy.* 2010;96(2):98-107.

**140.** Ir P, Horemans D, Souk N, Damme Wv. Using targeted vouchers and health equity funds to improve access to skilled birth attendants for poor women: a case study in three rural health districts in Cambodia. *BMC Pregnancy and Childbirth.* 2010;10(1):(7 January 2010).

**141.** Ekirapa-Kiracho E, Waiswa P, Rahman MH, Makumbi F, Kiwanuka N, Okui O, Rutebemberwa E, Bua J, Mutebi A, Nalwadda G, Serwadda D, Pariyo GW, Peters DH. Increasing access to institutional deliveries using demand and supply side incentives: early results from a quasi-experimental study. *BMC Int Health Hum Rights.* 2011;11 Suppl 1:S11.

**142.** Bellows NM, Bellows BW, Warren C. Systematic Review: the use of vouchers for reproductive health services in developing countries: systematic review. *Trop Med Int Health.* 2011a;16(1):84-96.

**143.** Smith KV, Sulzbach S. Community-based health insurance and access to maternal health services: evidence from three West African countries. *Soc Sci Med.* 2008;66(12):2460-2473.

**144.** Lu C, Chin B, Lewandowski JL, Basinga P, Hirschhorn LR, Hill K, Murray M, Binagwaho A. Towards universal health coverage: an evaluation of Rwanda Mutuelles in its first eight years. *PLoS ONE.* 2012;7(6):e39282.

**145.** Sekabaraga C, Diop F, Soucat A. Can innovative health financing policies increase access to MDG-related services? Evidence from Rwanda. *Health Policy Plan.* 2011;26 Suppl 2:ii52-62.

**146.** Long Q, Zhang T, Xu L, Tang S, Hemminki E. Utilisation of maternal health care in western rural China under a new rural health insurance system (New Co-operative Medical System). *Trop Med Int Health.* 2010;15(10):1210-1217.

**147.** Renaudin P, Prual A, Vangeenderhuysen C, Ould Abdelkader M, Ould Mohamed Vall M, Ould El Joud D. Ensuring financial access to emergency obstetric care: three years of experience with Obstetric Risk Insurance in Nouakchott, Mauritania. *Int J Gynaecol Obstet.* 2007;99(2):183-190.

**148.** Witter S, Arhinful DK, Kusi A, Zakariah-Akoto S. The experience of Ghana in implementing a user fee exemption policy to provide free delivery care. *Reprod Health Matters.* 2007;15(30):61-71.

**149.** Witter S, Adjei S, Armar-Klemesu M, Graham W. Providing free maternal health care: ten lessons from an evaluation of the national delivery exemption policy in Ghana. *Global Health Action.* 2009;2:1881.

**150.** Borghi J, Ensor T, Somanathan A, Lissner C, Mills A. Mobilising financial resources for maternal health. *Lancet.* 2006;368(9545):1457-1465.

**151.** Richard F, Witter S, de Brouwere V. Innovative approaches to reducing financial barriers to obstetric care in low-income countries. *Am J Public Health.* 2010;100(10):1845-1852.

**152.** Richard F, Ouedraogo C, Compaore J, Dubourg D, De Brouwere V. Reducing financial barriers to emergency obstetric care: experience of cost-sharing mechanism in a district hospital in Burkina Faso. *Trop Med Int Health.* 2007;12(8):972-981.

**153.** De Allegri M, Ridde V, Louis VR, Sarker M, Tiendrebeogo J, Ye M, Muller O, Jahn A. The impact of targeted subsidies for facility-based delivery on access to care and equity - Evidence from a population-based study in rural Burkina Faso. *J Public Health Policy.* 2012;33(4):439-453.

**154.** Hemminki E, Long Q, Zhang WH, Wu Z, Raven J, Tao F, Yan H, Wang Y, Klemetti R, Zhang T, Regushevskaya E, Tang S. Impact of Financial and Educational Interventions on Maternity Care: Results of Cluster Randomized Trials in Rural China, CHIMACA. *Matern Child Health J.* 2012.

**155.** Manandhar DS, Osrin D, Shrestha BP, Mesko N, Morrison J, Tumbahangphe KM, Tamang S, Thapa S, Shrestha D, Thapa B, Shrestha JR, Wade A, Borghi J, Standing H, Manandhar M, Costello AM. Effect of a participatory intervention with women's groups on birth outcomes in Nepal: cluster-randomised controlled trial. *Lancet.* 2004;364(9438):970-979.

**156.** Morrison J, Tamang S, Mesko N, Osrin D, Shrestha B, Manandhar M, Manandhar D, Standing H, Costello A. Women's health groups to improve perinatal care in rural Nepal. *BMC Pregnancy Childbirth.* 2005;5(1):6.

**157.** More NS, Bapat U, Das S, Alcock G, Patil S, Porel M, Vaidya L, Fernandez A, Joshi W, Osrin D. Community mobilization in Mumbai slums to improve perinatal care and outcomes: a cluster randomized controlled trial. *PLoS Med.* 2012;9(7):e1001257.

**158.** Azad K, Barnett S, Banerjee B, Shaha S, Khan K, Rego AR, Barua S, Flatman D, Pagel C, Prost A, Ellis M, Costello A. Effect of scaling up women's groups on birth outcomes in three rural districts in Bangladesh: a cluster-randomised controlled trial. *Lancet.* 2010;375(9721):1193-1202.

**159.** Baqui AH, El-Arifeen S, Darmstadt GL, Ahmed S, Williams EK, Seraji HR, Mannan I, Rahman SM, Shah R, Saha SK, Syed U, Winch PJ, Lefevre A, Santosham M, Black RE. Effect of community-based newborn-care intervention package implemented through two service-delivery strategies in Sylhet district, Bangladesh: a cluster-randomised controlled trial. *Lancet.* 2008;371(9628):1936-1944.

**160.** Kc NP, Kc A, Sharma N, Malla H, Thapa N, Aryal K, Vitrakoti R, Bhandari RM. Community participation and mobilization in community-based maternal, newborn and child health programmes in Nepal. *J Nepal Health Res Counc.* 2012a;9(2):101-106.

**161.** Lassi ZS, Haider BA, Bhutta ZA. Community-based intervention packages for reducing maternal and neonatal morbidity and mortality and improving neonatal outcomes. *Cochrane Database Syst Rev.* 2010(11):CD007754.

**162.** Bang AT, Bang RA, Reddy HM, Deshmukh MD, Baitule SB. Reduced incidence of neonatal morbidities: effect of home-based neonatal care in rural Gadchiroli, India. *J Perinatol.* 2005;25 Suppl 1:S51-61.

**163.** Woods DL. An innovative model of home-based neonatal care and research in India. *Pediatrics.* 2006;117(4):1425-1426.

**164.** Kumar V, Mohanty S, Kumar A, Misra RP, Santosham M, Awasthi S, Baqui AH, Singh P, Singh V, Ahuja RC, Singh JV, Malik GK, Ahmed S, Black RE, Bhandari M, Darmstadt GL. Effect of community-based behaviour change management on neonatal mortality in Shivgarh, Uttar Pradesh, India: a cluster-randomised controlled trial. *Lancet.* 2008;372(9644):1151-1162.

**165.** Bhandari N, Mazumder S, Taneja S, Sommerfelt H, Strand TA. Effect of implementation of Integrated Management of Neonatal and Childhood Illness (IMNCI) programme on neonatal and infant mortality: cluster randomised controlled trial. *BMJ.* 2012;344:e1634.

**166.** Pradhan YV, Upreti SR, Pratap KCN, K CA, Khadka N, Syed U, Kinney MV, Adhikari RK, Shrestha PR, Thapa K, Bhandari A, Grear K, Guenther T, Wall SN. Newborn survival in Nepal: a decade of change and future implications. *Health Policy Plan.* 2012;27 Suppl 3:iii57-71.

**167.** Mburu G, Iorpenda K, Muwanga F. Expanding the role of community mobilization to accelerate progress towards ending vertical transmission of HIV in Uganda: the Networks model. *J Int AIDS Soc.* 2012;15 Suppl 2:17386.

**168.** Darmstadt GL, Choi YJ, Arifeen SE, Sanwarul B, Rahman SM, Mannan I, Seraji HR, Winch PJ, Saha SK, Ahmed ASMNU, Ahmed S, Nazma B, Lee ACC, Black RE, Santosham M, Crook D, Baqui AH. Evaluation of a cluster-randomized controlled trial of a package of community-based maternal and newborn interventions in Mirzapur, Bangladesh. *PLoS ONE.* 2010(March):e9696.

**169.** Mullany LC, Lee CI, Paw P, Shwe Oo EK, Maung C, Kuiper H, Masenior N, Beyrer C, Lee TJ. The MOM Project: delivering maternal health services among internally displaced populations in eastern Burma. *Reprod Health Matters.* 2008;16(31):44-56.

**170.** Mullany LC, Lee TJ, Yone L, Lee CI, Teela KC, Paw P, Shwe Oo EK, Maung C, Kuiper H, Masenior NF, Beyrer C. Impact of community-based maternal health workers on coverage of essential maternal health interventions among internally displaced communities in eastern Burma: the MOM project. *PLoS Med.* 2010;7(8):e1000317.

**171.** Bhutta ZA, Memon ZA, Soofi S, Salat MS, Cousens S, Martines J. Implementing community-based perinatal care: results from a pilot study in rural Pakistan. *Bull World Health Organ.* 2008;86(6):452-459.

**172.** Bhutta ZA, Soofi S, Cousens S, Mohammad S, Memon ZA, Ali I, Feroze A, Raza F, Khan A, Wall S, Martines J. Improvement of perinatal and newborn care in rural Pakistan through community-based strategies: a cluster-randomised effectiveness trial. *Lancet.* 2011;377(9763):403-412.

**173.** Rosato M, Mwansambo C, Lewycka S, Kazembe P, Phiri T, Malamba F, Newell ML, Osrin D, Costello A. MaiMwana women's groups: a community mobilisation intervention to improve mother and child health and reduce mortality in rural Malawi. *Malawi Med J.* 2010;22(4):112-119.

**174.** Alam K, Tasneem S, Oliveras E. Performance of female volunteer community health workers in Dhaka urban slums. *Soc Sci Med.* 2012;75(3):511-515.

**175.** Mushi D, Mpembeni R, Jahn A. Effectiveness of community based Safe Motherhood promoters in improving the utilization of obstetric care. The case of Mtwara Rural District in Tanzania. *BMC Pregnancy Childbirth.* 2010;10:14.

**176.** Wallin L, Malqvist M, Nga NT, Eriksson L, Persson LA, Hoa DP, Huy TQ, Duc DM, Ewald U. Implementing knowledge into practice for improved neonatal survival; a cluster-randomised, community-based trial in Quang Ninh province, Vietnam. *BMC Health Serv Res.* 2011;11:239.

**177.** Nahar S, Banu M, Nasreen HE. Women-focused development intervention reduces delays in accessing emergency obstetric care in urban slums in Bangladesh: a cross-sectional study. *BMC Pregnancy Childbirth.* 2011;11:11.

**178.** Nahar T, Azad K, Aumon BH, Younes L, Shaha S, Kuddus A, Prost A, Houweling TA, Costello A, Fottrell E. Scaling up community mobilisation through women's groups for maternal and neonatal health: experiences from rural Bangladesh. *BMC Pregnancy Childbirth.* 2012;12:5.

**179.** Boone P, Mann V, Eble A, Mendiratta T, Mukherjee R, Figueiredo R, Jayanty C, Frost C, Padmanabh MR, Elbourne D. Community health and medical provision: impact on neonates (the CHAMPION trial). *BMC Pediatr.* 2007;7:26.

**180.** Lopes TC, Mota JA, Coelho S. Perspectives from a home based neonatal care program in Brazil's Single Health System. *Rev Lat Am Enfermagem.* 2007;15(4):543-548.

**181.** Sibley L, Buffington ST, Haileyesus D. The American College of Nurse-Midwives' home-based lifesaving skills program: a review of the Ethiopia field test. *J Midwifery Womens Health.* 2004;49(4):320-328.

**182.** Sibley L, Buffington ST, Tedessa L, Sr., McNatt K. Home-Based Life Saving Skills in Ethiopia: an update on the second phase of field testing. *J Midwifery Womens Health.* 2006;51(4):284-291.

**183.** Prata N, Ejembi C, Fraser A, Shittu O, Minkler M. Community mobilization to reduce postpartum hemorrhage in home births in northern Nigeria. *Soc Sci Med.* 2012;74(8):1288-1296.

**184.** Rahman A, Moran A, Pervin J, Rahman M, Yeasmin S, Begum H, Rashid H, Yunus M, Hruschka D, Arifeen SE, Streatfield PK, Sibley L, Bhuiya A, Koblinsky M. Effectiveness of an integrated approach to reduce perinatal mortality: recent experiences from Matlab, Bangladesh. *BMC Public Health.* 2011;11:914.

**185.** Fullerton JT, Killian R, Gass PM. Outcomes of a community- and home-based intervention for safe motherhood and newborn care. *Health Care Women Int.* 2005;26(7):561-576.

**186.** Skinner J, Rathavy T. Design and evaluation of a community participatory, birth preparedness project in Cambodia. *Midwifery.* 2009;25(6):738-743.

**187.** Marsh DR, Sternin M, Khadduri R, Ihsan T, Nazir R, Bari A, Lapping K. Identification of model newborn care practices through a positive deviance inquiry to guide behavior-change interventions in Haripur, Pakistan. *Food Nutr Bull.* 2002;23(4 Suppl):109-118.

**188.** Brugha R, Pritze-Aliassime S. Promoting safe motherhood through the private sector in low- and middle-income countries. *Bull World Health Organ.* 2003;81(8):616-623.

**189.** Ravindran TKS. Public-private partnerships in maternal health services. *Economic and Political Weekly.* 2011;46(48):43, 45-52.

**190.** Huntington D, Banzon E, Recidoro ZD. A systems approach to improving maternal health in the Philippines. *Bull World Health Organ.* 2012;90(2):104-110.

**191.** Kc A, Thapa K, Pradhan YV, Kc NP, Upreti SR, Adhikari RK, Khadka N, Acharya B, Dhakwa JR, Aryal DR, Aryal S, Starbuck E, Poudel D, Khanal S, Devekota MD. Developing community-based intervention strategies and package to save newborns in Nepal. *J Nepal Health Res Counc.* 2012;9(2):107-118.

**192.** Ha BT, Green A, Gerein N, Danielsen K. Health policy processes in Vietnam: a comparison of three maternal health case studies. *Health Policy.* 2010;98(2-3):178-185.

**193.** Tonmukayakul U, Velasco RP, Tantivess S, Teerawattananon Y. Lessons drawn from research utilization in the maternal iodine supplementation policy development in Thailand. *BMC Public Health.* 2012;12:391.

**194.** Hadley MB, Tuba M. Local problems; local solutions: an innovative approach to investigating and addressing causes of maternal deaths in Zambia's Copperbelt. *Reprod Health.* 2011;8:17.

**195.** Haththotuwa R, Senanayake L, Senarath U, Attygalle D. Models of care that have reduced maternal mortality and morbidity in Sri Lanka. *Int J Gynaecol Obstet.* 2012;119 Suppl 1:S45-49.

**196.** Rubayet S, Shahidullah M, Hossain A, Corbett E, Moran AC, Mannan I, Matin Z, Wall SN, Pfitzer A, Syed U. Newborn survival in Bangladesh: a decade of change and future implications. *Health Policy Plan.* 2012;27 Suppl 3:iii40-56.

**197.** Nair H, Arya G, Vidnapathirana J, Tripathi S, Talukder SH, Srivastava V. Improving neonatal health in South-East Asia. *Public Health.* 2012;126(3):223-226.

**198.** Khan A, Kinney MV, Hazir T, Hafeez A, Wall SN, Ali N, Lawn JE, Badar A, Khan AA, Uzma Q, Bhutta ZA. Newborn survival in Pakistan: a decade of change and future implications. *Health Policy Plan.* 2012;27 Suppl 3:iii72-87.

**199.** Koblinsky M, Matthews Z, Hussein J, Mavalankar D, Mridha MK, Anwar I, Achadi E, Adjei S, Padmanabhan P, Marchal B, De Brouwere V, van Lerberghe W. Going to scale with professional skilled care. *Lancet.* 2006;368(9544):1377-1386.

**200.** Zimba E, Kinney MV, Kachale F, Waltensperger KZ, Blencowe H, Colbourn T, George J, Mwansambo C, Joshua M, Chanza H, Nyasulu D, Mlava G, Gamache N, Kazembe A, Lawn JE. Newborn survival in Malawi: a decade of change and future implications. *Health Policy Plan.* 2012;27 Suppl 3:iii88-103.

**201.** Serruya SJ, Cecatti JG, Lago TG. [The Brazilian Ministry of Health's Program for Humanization of Prenatal and Childbirth Care: preliminary results]. *Cad Saude Publica.* 2004;20(5):1281-1289.

**202.** Enkhtuya B, Badamusuren T, Dondog N, Khandsuren L, Elbegtuya N, Jargal G, Surenchimeg V, Grundy J. Reaching every district - development and testing of a health micro-planning strategy for reaching difficult to reach populations in Mongolia. *Rural Remote Health.* 2009;9(2):1045.

**203.** Nigeria Federal Ministry of Health. Saving newborn lives in Nigeria: Newborn health in the context of the Integrated Maternal, Newborn and Child Health Strategy. 2nd edition. Abuja: Federal Ministry of Health, Save the Children, Jhpiego. *accessed at* [*http://www.healthynewbornnetwork.org/sites/default/files/resources/Nigeria%20Sit%20An%20final%20lowres_FINAL.pdf*](http://www.healthynewbornnetwork.org/sites/default/files/resources/Nigeria%20Sit%20An%20final%20lowres_FINAL.pdf)*.* 2011.

**204.** Orozco-Nunez E, Gonzalez-Block MA, Kageyama-Escobar LM, Hernandez-Prado B. [The experience of the Mexican maternal health care program Arranque Parejo en la Vida]. *Salud Publica Mex.* 2009;51(2):104-113.

**205.** Hernandez-Prado B, Kestler E, Diaz J, Walker D, Langer A, Lewis S, De la Vara-Salazar E, Melo-Zurita Mdel C, Iriarte E, Danel I, Donnay F, Aleman D, Serrano R, Morales E, Largaespada N, Gonzalez JD, Hernandez Mdel C, Mejia CE, Gonzalez G, Carrera Y, Valverde C, Luna R, Valencia-Mendoza A, Sosa-Rubi SG. [Situational profile and intervention strategy in the Mesoamerican region in maternal, neonatal and reproductive health area]. *Salud Publica Mex.* 2012;53 Suppl 3:S312-322.

**206.** Jashi M, Viswanathan R, Ekpini R, Chandan U, Idele P, Luo C, Legins K, Chatterjee A. Informing policy and programme decisions for scaling up the PMTCT and paediatric HIV response through joint technical missions. *Health Policy Plan.* 2012.

**207.** Uwimana J, Zarowsky C, Hausler H, Jackson D. Engagement of non-government organisations and community care workers in collaborative TB/HIV activities including prevention of mother to child transmission in South Africa: opportunities and challenges. *BMC Health Serv Res.* 2012;12:233.

**208.** Kabakian Khasholian T, El Kak F, Shayboub R. Birthing in the Arab region: translating research into practice. *East Mediterr Health J.* 2012;18(1):94-99.
